# Machine learning-based test smell detection

**DOI:** 10.1007/s10664-023-10436-2

**Published:** 2024-03-05

**Authors:** Valeria Pontillo, Dario Amoroso d’Aragona, Fabiano Pecorelli, Dario Di Nucci, Filomena Ferrucci, Fabio Palomba

**Affiliations:** 1 Software Engineering (SeSa) Lab - University of Salerno, Fisciano, Italy; 2 Software Languages (Soft) Lab - Vrije Universiteit Brussel, Brussel, Belgium; 3 Tampere University, Tampere, Finland

**Keywords:** Test smells, Test code quality, Machine learning, Empirical software engineering

## Abstract

Test smells are symptoms of sub-optimal design choices adopted when developing test cases. Previous studies have proved their harmfulness for test code maintainability and effectiveness. Therefore, researchers have been proposing automated, heuristic-based techniques to detect them. However, the performance of these detectors is still limited and dependent on tunable thresholds. We design and experiment with a novel test smell detection approach based on machine learning to detect four test smells. First, we develop the largest dataset of manually-validated test smells to enable experimentation. Afterward, we train six machine learners and assess their capabilities in within- and cross-project scenarios. Finally, we compare the ML-based approach with state-of-the-art heuristic-based techniques. The key findings of the study report a negative result. The performance of the machine learning-based detector is significantly better than heuristic-based techniques, but none of the learners able to overcome an average F-Measure of 51%. We further elaborate and discuss the reasons behind this negative result through a qualitative investigation into the current issues and challenges that prevent the appropriate detection of test smells, which allowed us to catalog the next steps that the research community may pursue to improve test smell detection techniques.

## Introduction

Test cases are the first barrier against software faults, particularly during regression testing (Myers et al. 2011). Development teams rely on their outcome to decide whether it is worth merging a pull request (Gousios et al. 2015) or even deploying the system (Beller et al. 2017). At the individual level, the developer’s productivity is also partially dependent on the ability of tests to find real defects in production code (Zhang and Mesbah 2015) and the timely diagnosis of the underlying causes (Perez et al. 2017). Unfortunately, when developing test cases, programmers may apply sub-optimal implementation choices that could introduce test debt (Kruchten et al. 2012), namely potential design problems that lead to unforeseen testing and debugging costs for developers (Maldonado and Shihab 2015). Test smells, i.e., symptoms of poor design or implementation choices in test code (Van Deursen et al. 2001), represent one of the most significant sources of test debt (Samarthyam et al. 2017; Tufano et al. 2016). Several empirical studies in the recent past have focused on test smells to understand their properties (Tufano et al. 2016) and their impact on maintainability (Spadini et al. 2018; Bavota et al. 2015; Grano et al. 2019) and test effectiveness (Grano et al. 2019), by showing compelling evidence of the risks associated with the presence of test smells for software dependability and test code quality.

For these reasons, researchers have investigated methods for automatically detecting test smells (Garousi and Küçük 2018). Such techniques discriminate tests affected (or not) by a particular type of smell by applying detection rules that compare the values of relevant metrics extracted from test code against some empirically identified thresholds. For instance, Van Rompaey et al. (2007) proposed a metric-based technique that computes several structural metrics (e.g., number of production code calls made by a test case) and combines them into detection rules to highlight the likelihood of a test being smelly. A test is marked as smelly if the value overcomes a threshold. Despite the effort spent by researchers so far, existing test smell detectors still suffer from two key limitations. First and foremost, they have limited detection capabilities, behaving similarly to a random guessing approach (Van Rompaey et al. 2007; Greiler et al. 2013; Palomba et al. 2018). Second, their performance is strongly influenced by the thresholds used in the detection rules to discriminate between smelly and non-smelly tests (Fernandes et al. 2016; Garousi and Küçük 2018). These restrictions threaten the practical applicability of these approaches. Machine learning represents one of the possible solutions to the limitations mentioned above. Besides avoiding the need to combine metrics using detection rules, a machine learning approach would avoid the problem of selecting thresholds, thus representing a promising solution to alleviate the limitations of heuristic-based techniques.

In this paper, we aim to build on top of the existing knowledge, exploring the capabilities of machine learning to improve the performance of existing test smell detectors through an empirical investigation. More specifically, the proposed approach employs structural and textual metrics as features to estimate the likelihood of a test being smelly and is instantiated for the detection of four test smell types, i.e., *Eager Test*, *Mystery Guest*, *Resource Optimism*, and *Test Redundancy*. Afterward, we empirically evaluate the performance of the devised detector on a new dataset of *Java* projects—which we manually build, publicly releasing the largest manually-crafted dataset of test smells to date (Garousi and Küçük 2018)—and compare its performance with three state-of-art heuristic-based techniques. The findings of our study can be configured as a negative result. The machine learning approach performs better than the traditional, heuristic-based techniques but it is ineffective when detecting all test smells.

As a consequence of our negative result, we conduct a qualitative investigation into the issues and challenges that prevent the proper identification of test smells. Such a qualitative investigation allows us to elicit and catalog the root causes of failures of machine learning- and heuristic-based detectors, providing the research community with insights and practical examples of when and why current test smell detectors fail, other than how to improve the currently available instruments. We specifically identify several issues related to the inaccurate definition of test smells, improper analysis and measurement of the characteristics of those smells and inappropriate treatment of corner cases. Based on our qualitative analysis, we finally outline take-away messages and actionable insights for future research in the field.

*Structure of the Paper* Section 2 overviews the related literature and explains how we advance the state of the art. In Section 3, we elaborate on the research questions driving our study, while Section 4 reports the method used to define the novel test smell dataset. The machine learning approach to detecting code smells is discussed in Section 5, while its empirical evaluation is reported in Section 6 and discussed in Section 7. The potential limitations of the study are reported in Section 8, other than the mitigation strategies applied. Finally, Section 9 concludes the paper and outlines our future research agenda.

## Related Work

Investigations on the design of test code were initially pointed out by Beck (2003). Van Deursen et al. (2001) and Meszaros (2007) defined catalogs of test smells along with their refactoring actions. More recently, Greiler et al. (2013) devised TestHound, a heuristic-based approach to identify six test smell types evaluated through semi-structured interviews. Palomba et al. (2018) devised Taste, a test smell detector that leverages textual metrics (e.g., the conceptual cohesion of test methods Marcus and Poshyvanyk 2005) to complement previous techniques and identify three test smell types. The detection rules proposed by Palomba et al. (2018) were later implemented in Darts (Lambiase et al. 2020), an Intellij plugin that makes Taste usable through a user interface. Peruma et al. (2020) proposed tsDetect, a test smell detector that identifies 19 test smell types, including *Assertion Roulette*, *Eager Test*, and *Lazy Test*. Maier and Felderer (Maier and Felderer 2023) recently introduced SniffTest, a test smell detector based on language analysis methods to identify instances of five test smell types such as *Anonymous Test*, *Long Test*, *Conditional Test Logic*, *Assertion Roulette*, and *Rotten Green Test*. Pecorelli et al. (2020) implemented VITRuM, a Java plugin to provide developers with static and dynamic test-related metrics and identify seven test smell types. Similarly, Wang et al. (2021) proposed PyNose, a Python plugin to detect 17 test smells. Koochakzadeh and Garousi (2010) designed TeReDetect, a tool that uses rules and dynamic metrics to detect *Test Redundancy*, i.e., a test that could be removed without impacting the test suite. De Bleser et al. (2019) proposed SoCRATES, a fully automated tool that combines syntactic and semantic data to identify six test smells in Scala software systems. Our paper is complementary to this research since it introduces an orthogonal method based on machine learning to identify test smells; compared to previous work, the proposed approach would not require tuning thresholds and may be designed to combine multiple metrics previously employed in isolation. Furthermore, we conduct a large-scale empirical study on a manually-validated dataset, making our investigation the largest in test smell detection research.

Other related work concerns the empirical analyses of test smells. Tufano et al. (2016) investigated the lifecycle of test smells, while Bavota et al. (2015) showed that test smells are highly diffused in software projects and impact the understandability of test code. Similar results were later confirmed (Martins et al. 2023) and achieved when considering automatically generated test cases (Grano et al. 2019) and in software systems developed using the combination of Scala and ScalaTest  (De Bleser et al. 2019). In addition, Rwemalika et al. (2023) investigated test smells in interactive user test cases, finding that these are highly diffused and potentially harmful. Furthermore, Spadini et al. (2018) showed that test smells impact the maintainability of both test and production code. Spadini et al. (2019) also discovered that test-driven code reviews might help developers discover design flaws in test code. All these studies serve as motivation for our paper. Based on the empirical evidence provided in the past, test smells represent a relevant threat to software reliability that should be promptly detected. We aim to employ machine learning (ML) algorithms previously used for code smell detection—the interested reader may find a comprehensive literature analysis on machine learning for code smell detection by Azeem et al. (2019). Although code and test smells share a similar high-level definition, they do not share the same characteristics. It is, therefore, worth analyzing the main differences we expect compared to the previous research on code smell detection. According to the literature available, ML-based code smell detection comes with three significant limitations concerning (i) data imbalance, (ii) subjectivity of code smell data, and (iii) a set of predictors that poorly contribute to the accuracy of the detection (Pecorelli et al. 2019b).

As for the data imbalance limitation, previous literature has shown that test smells are more diffused than code smells, e.g., Bavota et al. (2015) found *Eager Test* instances to affect around 35% of test classes. Conversely, code smells typically affect a meager percentage of classes (i.e., around 2%) (Palomba et al. 2016). Therefore, it is reasonable to believe that the limitation of data imbalance could have a lower significance when dealing with test smells. Nevertheless, in the context of our empirical study, we investigate the use of data balancing to understand whether this additional step could benefit the models.

Concerning subjectivity, we envision a strong relationship between test and code smells—this was already shown by Tufano et al. (2016). The manually-validated dataset discussed in Section 4 may have suffered from the subjectivity of the authors who made the validation; in response, we also involved external developers to double-check the manual validations performed when building the dataset. As for the predictors, we rely on metrics adopted by existing heuristic techniques to verify the contributions provided by those metrics, other than identifying potential limitations resulting from their adoption.

## Goals and Research Questions

The *goal* of the study was to evaluate the suitability of machine learning for test smell detection, with the *purpose* of improving test code quality through the removal of detrimental design flaws. The *perspective* is of researchers and practitioners interested in understanding the performance and limitations of machine learning techniques for test smell detection. Specifically, our paper was structured around three research questions (**RQ**s), namely:

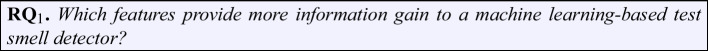

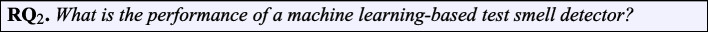

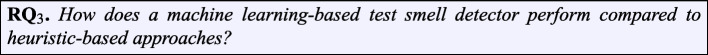







With the first research question (**RQ**_1_), we sought to understand which metrics contribute the most to detecting test smells. These observations were used to (i) quantify the predictive power of metrics and (ii) identify the most promising features to include in our machine-learning approach. In **RQ**_2_, we run our machine learning approach against a manually validated oracle of test smells (built according to the operations reported in Section 4) to quantify its detection performance capabilities. Afterward, with **RQ**_3_, we aimed to compare the performance of our technique with the one achieved by state-of-the-art approaches based on heuristics: Such validation allowed us to understand the actual value of a machine learning approach, i.e., should it work worse than heuristic approaches, its usefulness would be limited, as practitioners might still find heuristic approaches more beneficial.

To design and report our empirical study, we followed the empirical software engineering guidelines by Wohlin et al. (2012), other than the *ACM/SIGSOFT Empirical Standards*.^1^

## Dataset Construction

Creating a manually-validated dataset of test smells represented the first step of our investigation. This step included selecting projects and test smell types, besides the manual data collection to build the dataset. The following sections report on each of these points.

### Projects Selection

We collected test data from a dataset of 66 open-source Java projects, publicly available on GitHub, and 51,549 test cases. These projects are part of a larger, popular dataset known as the International Dataset of Flaky Tests (IDoFT).^2^ The selection was driven by two main factors. First, we considered the entire set of test cases contained in these projects, i.e., not only those labeled as flaky, to complement IDoFT with additional information related to test smells. In this way, researchers might have been provided with a unique database containing various test code-related issues, which would be beneficial to stimulate further research on test code quality. These projects were highly diverse in terms of scopes and sizes, hence representing an ideal source to mitigate possible threats to external validity—our online appendix provides detailed statistics on those projects (Pontillo et al. 2023). Second, the rationale for using this dataset came from previous observations made by Pontillo et al. (2021, 2022). In their study, the authors ran a state-of-the-art test smell detector named VITRuM (Pecorelli et al. 2020) and identified a high number of test smells, i.e., they found that around 80% of test cases were smelly. While we did not use automated tools to collect test smell data, the high diffuseness of test smells in the dataset suggested that it may be worth manually analyzing those projects—as documented in the next sections, this resulted in a reasonable choice, as we found that the percentage of test smells validated as smelly by both us and VITRuM for *Eager Test*, *Mystery Guest*, and *Resource Optimism* was 66%, 36%, and 7%, respectively.

### Test Smell Selection

In the context of our work, we needed to experiment with test smells detectable using machine learning algorithms. In addition, we aimed to compare the performance of those algorithms with the ones of state-of-the-art heuristic tools. As such, we needed to identify a set of test smells that would have allowed us to meet two requirements: (1) their detection should have been based on at least two metrics—if a test smell can be detected through an individual metric, it would not have made sense to experiment with machine learning solutions as this would have contributed to neither **RQ**_1_ and **RQ**_2_; (2) their detection should have been supported by at least one tool—otherwise, we could not have addressed **RQ**_3_. Based on these requirements, we first performed a comprehensive literature analysis to extract all the test smells automatically detectable by the current techniques. We started from the list of test smell detection tools reported in a systematic mapping study by Aljedaani et al. (2021). This study reports all the test smell detection tools available in the literature and the test smells they detect. From an initial set of 22 tools, we included only those (i) supporting Java as a programming language, as the vast majority of tools use only Java as the target language, and (ii) relying on a metric-based approach, since machine learning classifiers require a set of metrics to be used as predictors. Specifically, we excluded three tools that do not support Java as a target programming language and nine tools that do not rely on a metric-based approach to detect test smells. This filtering phase led us to a final number of ten tools.

Afterward, we analyzed each tool and extracted information about the test smells they detect and the metrics they use for the detection. We extracted a total number of 31 different test smells. We further considered only the test smells for which at least two metrics have been defined (more details about the metrics are reported in Section 4.2), leading us to select a set of six test smell types, namely *Empty Test*, *Eager Test*, *Mystery Guest*, *Sensitive Equality*, *Resource Optimism*, and *Test Redundancy*. We discarded 25 of them because their detection was based only on a single metric. It is important to note that, in this case, the second requirement (i.e., the detection must be supported by at least one tool) is intrinsically guaranteed since we extracted only the smells that are detected by the ten selected tools—more details are reported in out online appendix (Pontillo et al. 2023).

However, we noticed that detecting two of these smells was very trivial (i.e., *Empty Test* and *Sensitive Equality*); therefore, the use of a machine learning-based approach would not lead to any detection performance improvement other than being an overkill in terms of computational costs.

*Empty Test* is defined as *“a test method that is empty or does not have executable statements”*; thus, a heuristic approach could objectively identify test cases that suffer from this issue. As a proof of that, Peruma et al. (2020) applied this heuristic to detect *Empty Test* instances within TsDetect, obtaining an F-Measure of 100%. The same consideration applies to *Sensitive Equality*, which occurs when *“an assertion has an equality check by using the toString method”*. Two existing heuristic-based detectors, namely TsDetect (Peruma et al. 2020) and the one introduced by Bavota et al. (2012), are able to detect *Sensitive Equality* instances with high accuracy. In particular, TsDetect (Peruma et al. 2020) detects a test method as smelly if it invokes the toString method of an object, while the detector by Bavota et al. (2012) verifies that a toString method of an object is called within an assertion. According to the performance reported within these previous papers, TsDetect (Peruma et al. 2020) reaches an F-Measure of 90%, while Bavota et al. (2012) claimed an F-Measure of 100%. Based on the above consideration, we decided to discard these two test smells, resulting in a final set of four test smells reported in Table 4 together with their definition.

Another discussion point concerns the *Resource Optimism* smell. Given its definition, it is likely that information-flow or dynamic analyses might be potentially more suitable for detecting it. In this sense, a machine learning solution might be sub-optimal, yet we aimed to assess the extent to which it may provide valuable insights to detect the smell. These observations might be used to understand how the performance of machine learning compares to existing approaches and, perhaps, be later used by researchers to combine it with novel, more precise information flows or dynamic sources of information.

### Test Smell Data Collection

Once we had selected projects and test smell types, we then proceeded with the manual test smell classification. The first two authors of the paper acted as the “inspectors” to mitigate potential subjectiveness issues due to a single inspector performing the manual validation. The other authors were also involved whenever needed, as further discussed later in this section. For the sake of transparency, it is worth remarking that the authors involved have 3 to 15 years of experience on themes connected to test code quality, test smells, and empirical software engineering. In addition, most of the authors were also experienced in devising manually-built datasets. Overall, the amount of effort required by the dataset-building phase was quantified in 320 hours/person.

Given the impracticability of manually analyzing all 51,549 test cases, the process was conducted on a statistically significant stratified sample of 9,633 test cases (confidence level = 99%, margin of error = 1%). When defining the sample, we used the distribution of test cases per software project as stratification criterion. In this way, we could analyze a sample that kept the same proportion of test cases of the original population, i.e., a larger project will account for more tests than a smaller one. It is worth pointing out that we could not take the distribution of test smells into account when sampling the initial population of test cases, as the sample was built exactly for the sake of manually detecting test smells. Indeed, the idea of sampling the initial population of test cases came from our willingness to assess the smelliness of test cases manually —in other terms, when sampling the population we did not have information about test smells - this was indeed the intended result of the manual validation. After defining the sample, we proceeded with the actual validation, which was approached through a three-step process—Table 1 reports the number of test cases analyzed at each stage:

**Table 1:** Number of test cases analyzed at each stage of the validation process

	Inspector #1	Inspector #2	200 external practitioners
#1 Initial Validation	963 test cases		
#2 Internal Validation	4335 test cases	4335 test cases	
#3 External Validation			480 test cases

*Step #1: Initial Validation* As a first step, both inspectors independently analyzed a subset of 963 test methods (equal to 10% of the total)—a third inspector (i.e., the third author of the paper) was in charge of making the final decision about the disagreements. Specifically, the tasks performed by the two inspectors are elaborated in the following:

1. They consider each test method they were assigned to, opening the corresponding code in their preferred IDE, i.e., they were both IntelliJ users.
2. By taking the definitions of the test smells considered in our work, they assessed whether the test code was affected by any of them. The inspectors were allowed to navigate the code as they liked so that they could assess the test method on its own and how it interacted with other test or production methods. They could also rely on additional data, e.g., project documentation, contribution guidelines, or developer’s discussion, to acquire contextual information and more appropriately assess the smelliness of the test method.
3. They filled a spreadsheet that was designed to have six columns: the first, named *‘Test Method’*, took track of the name (and path) of the test method analyzed; the second to fifth columns, named *Eager Test*, *‘Mystery Guest’*, *‘Resource Optimism’*, and *‘Test Redundancy’*, respectively, stored boolean values representing whether the test method contained or not each of the considered test smells; finally, the last column, named *‘Notes’*, was included to let the inspectors write down notes and observations that might be useful for the subsequent validation steps.

Upon completion, the results of this first validation were compared through Cohen’s $\kappa$ (Cohen 1960), which measures the *inter-rater agreement* of the inspection task. As an outcome, the two inspectors reached an agreement of 0.76, which indicates a *substantial* agreement (McHugh 2012). The inspectors, including the third one, also scheduled an online meeting to discuss the validation process, the cases of disagreement, the challenges they faced, the annotations reported in the *‘Notes’* field of the spreadsheet, and how they dealt with corner cases. The meeting was performed through Skype and lasted 1.5 hours. The result of the meeting was instrumental for the second step, as it allowed the inspectors to do a retrospective and set a baseline.

*Step #2: Internal Validation* As a second step, the unclassified instances were equally split between the two inspectors, reiterating the same tasks described above. Upon completion of the validation, we scheduled two meetings. In the first, the three inspectors mainly involved in the process met again to discuss further the operations performed. This meeting was performed on Skype and lasted 1.5 hours. In the second, more formal, all the authors of the paper in which the specific actions conducted during the inspection process were critically reviewed to discover possible inconsistencies in the way the inspectors conceptually classified test smell instances. The meeting was hybrid (the remote component was realized through Skype) and lasted 3 hours. As an outcome of the meeting, we decided to perform an additional round of cross-check validation: each of the two main inspectors involved in the process double-checked the validations made by the other to increase the robustness of the dataset. As a result of the cross-check, the Cohen’s $\kappa$ measured 0.84, indicating an *almost perfect* agreement (McHugh 2012).

*Step #3: External Validation* While the formal process described above was supposed to mitigate possible bias when labeling the smelliness of test code, this may still contain subjective test smell instances. For this reason, we planned an external validation of the test smells included in the dataset, which involved experienced software testers. We approached such an external validation as a *coherence check* of the internal validation rather than as an extensive assessment thereof—indeed, the external validation must be seen as a mitigation of the possible subjectivity bias affecting the internal validation. The goal was to assess the extent to which external practitioners would label the smelliness of test cases similarly to the internal validation: in the positive case, this coherence check would have highlighted the soundness of the internal validation procedure, other than the reliability of the dataset constructed in our work. Since it was unreasonable to ask for an external validation of the entire set of 9,633 test cases (it would have been excessively costly in terms of time and effort required by external developers), we randomly selected a subset of 480 test cases (around 5% of the test cases considered). There are some observations to make in terms of the sampling strategy and its impact. We preferred a random selection as opposed to a stratification based on the distribution of the test smells identified during our manual validation. In this case, the rationale was to let practitioners validate test cases having different properties according to their own experience with the aim of challenging and/or corroborating our own validation. On the one hand, the validation of random samples might have led practitioners to identify false negatives of the internal validation, i.e., instances labeled by us as non-smelly and by practitioners as smelly-this would have potentially imposed another round of internal, manual validation. On the other hand, practitioners might have assessed a random sample of test cases labeled as smelly during the internal validation, providing indications on the soundness of the operations performed by the inspectors. A distribution-aware selection solely looking at the distribution of the test cases labeled as smelly could have not reached the same result, as we would have not selected test cases labeled as non-smelly in our internal validation, hence possibly missing information on false negatives. In any case, it is worth reporting that the random sample still kept a similar proportion of the test smells within the validation set. We indeed had 127 *Eager Test* instances (26% of the test cases of the sample), 68 *Mystery Guest* instances (14%), 31 *Resource Optimism* instances (6%), and 2 *Test Redundancy* instances (0.4%); in addition, 252 test cases (53%) were labeled as non-smelly in the internal validation. In the sampled population, *Eager Test* instances represented 28% of all test cases, *Mystery Guest* instances formed the 16% of the test cases, *Resource Optimism* instances the 8%, and *Test Redundancy* the 0.4%, with the non-smelly tests representing 48% of all test cases—Table 3 reports information on the diffuseness of smelliness in the sample. In other terms, the random sample did not negatively impact the representativeness of smelly and non-smelly test cases.

**Table 2:** List of questions for the background part in the survey with the type of response provided

Section 1: Participant’s background		Type
#1	What kind of developer are you?	Multiple choice (Industrial, Open-source, Startup, Student, Researcher)
#2	How many years of experience do you have with the Java programming language?	Paragraph
#3	Please rate your level of expertise with the Java programming language.	5-point Likert scale
#4	How many years of experience do you have in Software Testing?	Paragraph
#5	To what extent do you perform each of the following types of testing in your projects?	Multiple-choice grid (Unit, Integration, System, Acceptance, Usability testing from “Never” to “Frequently”)
#6	How familiar are you with the concept of test smells, i.e., symptoms of sub-optimal design choices adopted when developing test cases?	5-point Likert scale

Table 2 reports questions related to the participant’s background. In particular, we asked for information on the context in which participants usually developed, e.g., industrial or academic, their knowledge of the Java programming language, how much and which testing they typically do when developing, and their familiarity with test smells.

We involved 200 external developers through the Prolific platform,^3^ a research instrument to select research participants. To mitigate the possible self-selection or voluntary response bias, we introduced a monetary incentive of 9 USD. Incentives are well-known to mitigate self-selection or voluntary response bias, other than increasing the response rate, as shown in previous studies targeting the methods to increase response rate in survey studies (Heckman 1990; Sakshaug et al. 2016). By setting the appropriate filters, we involved practitioners working in IT. More specifically, the developers were provided with a definition of the test smells subject of the study and asked to assess the smelliness of four test cases, i.e., the external developers performed very similar tasks as the inspectors in the internal validation, allowing us to compare the outcomes produced fairly. The four test cases to show to practitioners were randomly selected from the sample of 480 test cases, which means that they may have dealt with either one or more smelly or non-smelly test cases. The choice of limiting the amount of test cases to assess to four was dictated by two main reasons. First and foremost, we aimed at limiting the cognitive load required by practitioners to perform the task: we deemed four test cases a reasonable amount to let practitioners be focused on the task and provide us with reliable insights - a higher number of test cases might have negatively impacted the cognitive load, possibly biasing the external validation. Second, our choice was motivated by the willingness to take the survey short, which is a relevant factor impacting the response rate of survey studies: we designed the external validation to be conducted within 10 minutes-including both answers to background questions and validation of the four test cases. A longer study involving the validation of more test cases may have lowered participation, affecting the validity of the external validation. Note that, having 480 tests and 200 developers, we could also perform cross-checking, i.e., several developers assessed a subset of 262 test cases to verify the consistency among the evaluations provided.

Upon completing the data collection, we first filtered out 16 answers from developers with less than one year of experience in testing—we considered them not experienced enough to provide reliable insights. Regarding demographic details, we analyzed the data collected directly from Prolific, looking at the self-declared information made available by the participants. We focused on Age, Nationality, Language, and Sex. In terms of age, the lowest age is 18, while the highest age is 62. The median is 26. Analyzing the nationality, 66% of respondents came from Europe, 19% from Africa, 13% from Asia, and 2% from America. English and Portuguese are the most common languages spoken by the participants (each for 32%), while other languages such as Italian, German, Greek, etc. contribute between 5% and 1%. Finally, 77% of the participants are male, while 23% are female—we reported all data anonymously in the online appendix (Pontillo et al. 2023). As for the other 184 responses, 81% of the participants have more than three years of software development experience, and 53% have more than three years of experience with Java. Almost 37% of the participants have more than three years of software testing experience, and 50% of the practitioners declared that they perform unit testing *frequently*— more details about the participants’ background and their experience with software development and testing are reported in our online appendix (Pontillo et al. 2023).

**Table 3:** Diffuseness of test smells in the dataset used for the external validation (480 test cases) and in the entire dataset (9,633 test cases). We reported the various combinations of test smells and non-test smells present in the datasets. The first row represents test cases that are no-smelly, the last row represents the test cases with all four test smells analyzed, and the rows in between are all combinations

Test red.	Res. opt.	Mystery guest	Eager test	Total	
				Ext. valid.	Entire dataset
0	0	0	0	307	5,976
0	0	0	1	103	2,082
0	0	1	0	22	413
0	0	1	1	14	391
0	1	0	0	0	3
0	1	0	1	0	0
0	1	1	0	23	513
0	1	1	1	8	207
1	0	0	0	0	17
1	0	0	1	1	13
1	0	1	0	0	0
1	0	1	1	1	3
1	1	0	0	0	0
1	1	0	1	1	0
1	1	1	0	0	7
1	1	1	1	0	0

Afterward, we assessed the consistency of the answers provided by developers: on average, for each test smell instance, 88% of participants assessed it in the same manner. This result looks interesting, especially when compared to the existing body of knowledge that assessed the developer’s perception of test smells (Tufano et al. 2016). Our findings suggest that developers’ awareness of test code quality issues may increase when providing them with specific definitions of test smells—we plan to further investigate this matter as part of our future research agenda. Finally, we computed the Cohen’s $\kappa$ coefficient between the evaluations provided by the inspectors on the sample instances and the evaluations provided by the majority of the developers in the survey study—in other terms, in the case an instance was evaluated differently by different developers, we applied a majority voting strategy to identify the most popular evaluation of that instance. The Cohen’s $\kappa$ measured 0.67, indicating a *good* agreement (McHugh 2012). We did not observe any case where the developer’s recommendations drastically differed from those performed by the inspectors; therefore, we did not change the original classification. The results obtained from this external validation were deemed sufficient to address the question about the potential bias of the internal validation; as such, we considered the dataset construction phase concluded.

The process described above led to the creation of the most extensive test smell dataset up to date—Table 3 reports details on the diffuseness of smelliness in the dataset. We obtained 2,699 instances of *Eager Test* (of which 2,082 test cases have only this smell), 1,534 instances of *Mystery Guest* 413 istances present only *Mystery Guest as test smell*, 730 instances of *Resource Optimism* (of which only three test cases have only this smell), and 40 instances of *Test Redundancy* (17 instances are pure *Test Redundancy*). We publicly released the dataset in our appendix (Pontillo et al. 2023). Besides indicating the smelliness of each test smell, we also released the anonymized evaluations received by the developers. We hope this dataset will be helpful to test code quality researchers to investigate further both test smell detectors and the developer’s awareness of test quality concerns.

## Machine Learning-based Test Smell Detection

We illustrate the approach employed to develop and experiment with a machine learning-based approach for test smell detection.

*Dependent Variable* As we aimed at automatically detecting the presence of test smells, the dependent variable is a binary value indicating the presence/absence of a specific test smell type. We considered the outcome of the validation process discussed in Section 4 as a dependent variable.

*Independent Variables* To collect a set of reliable predictors for each test smell under consideration, we used the metrics from heuristic approaches already available in the literature—the identification of new features was not in the scope of our investigation. Specifically, while performing the process described in Section 4 to select test smells, we collected all the metrics defined and used by the available detection approaches. Table 4 reports the list of metrics used for classifying each test smell with their description. We used these metrics as features to learn the machine learning algorithms. Our online appendix (Pontillo et al. 2023) also includes references to all the tools relying on the same metrics.

**Table 4:** Test smells included in our study, their definition, and the independent variables for each smell under investigation

Test smell	Definition	Metric	Description	Structural/textual
Eager test	A test method involving many methods of the object being tested.	NMC	Number of method calls	Structural
		TMC	Test method cohesion, i.e., the average textual similarity between all the pairs methods called by a test method	Textual
		TS	Textual scattering, i.e., the extent to which the text within the method body is conceptually scattered	Textual
		NRF	Number of references to files	Structural
Mystery guest	A test that uses external resources (e.g., databases or files).	NRDB	Number of references to database	Structural
Resource optimism	A test that uses external resources without checking the state of these.	ERNC	State of external resources, which are not files, not checked	Structural
		FRNC	State of file resources not checked	Structural
		PR	Pair redundancy is the ratio between the items covered by a test and those covered by another one	Structural
Test redundancy	A test that could be removed without impacting the test suite.	SR	Suite redundancy is the ratio between the items covered by a test compared and those covered by all other tests in the test suite	Structural

*Selecting Machine Learning Algorithms* To the best of our knowledge, our work investigates the first machine learning-based test smell detector; therefore, the most suitable classifier is still unknown. We have experimented with a set of classifiers belonging to different families that have been widely used in problems related to software maintenance and evolution (Catolino et al. 2018; Catolino and Ferrucci 2019; Catolino et al. 2019; Di Nucci et al. 2017; Pecorelli et al. 2019a, b). The goal of such extensive experimentation was to (i) understand which machine learning algorithm was the best for test smell detection and (ii) increase the generalizability of the results. More specifically, we assessed the capabilities of *Decision Tree* (Freund and Mason 1999), *Naive Bayes* (Duda and Hart 1973), *Multilayer Perceptron* (Taud and Mas 2018), and *Support Vector Machine* (Noble 2006), as basic classifier. We also considered two ensemble techniques, such as *Ada Boost* (Schapire 2013) and *Random Forest* (Breiman 2001).

*Model Configuration and Training* When training the selected machine learners, we experimented with multiple under- and over-sampling techniques to balance our data to understand how those algorithms may improve the test smell detection capabilities. As for the under-sampling, we considered the use of *NearMiss 1*, *NearMiss 2*, and *NearMiss 3* algorithms (Yen and Lee 2006). These compute the distance between instances of the majority and minority classes. Then, the algorithms select the instances of the majority class that have the shortest distance from instances of the minority class and remove them. The underlying idea is that removing the most similar majority samples increases the diversity of the training set and, therefore, lets a machine learner more appropriately learn features. We also experimented with a *Random Undersampling* approach, which randomly explores the distribution of majority instances and under-samples them. As for the over-sampling, we investigated *Synthetic Minority Over-sampling Technique*, a.k.a *SMOTE* (Chawla et al. 2002), and advanced versions of this algorithm, i.e., *Adaptive Synthetic Sampling Approach*, a.k.a *ADASYN* (He et al. 2008) and the *Borderline-SMOTE* (Han et al. 2005). While the basic *SMOTE* uses a simple k-nearest neighbor function to identify the minority class instances to over-sample, *ADASYN* over-samples the instances according to their learning difficulty. Instead, *Borderline-SMOTE* selects the minority class instances based on their similarity compared to the majority class instances. We also experimented with a Random Oversampling approach, which randomly explores the distribution of the minority class and over-samples them.

Finally, concerning the classifiers configuration, we experimented with the hyper-parameters of the classifiers using the *Random Search* strategy (Bergstra and Bengio 2012): this search-based algorithm randomly samples the hyper-parameters space to find the best combination of hyper-parameters maximizing a scoring metric (i.e., the Matthews Correlation Coefficient). We developed the entire pipeline with the Scikit-Learn library (Pedregosa et al. 2011) in Python.

*Model Validation* To assess the performance of our models, we performed both within- and cross-project validation. These validations aimed to quantify the performance of the models in two different scenarios. We were indeed interested to understand (i) how accurate can the performance be when a test smell detection model was trained using data of the same project where it should be applied and (ii) how accurate the model was when trained using external data to the project where it should be applied. For the within-project validation, we performed a stratified ten-fold cross-validation (Stone 1974) for each project. This strategy randomly partitions the data into ten folds of equal size, allowing us to maintain the correct proportion in every split between smelly and non-smelly instances. It iteratively selects a single fold as a test set while the other nine are used as a training set. For the cross-project validation, we adopted the *Leave-One-Out Cross-Validation* strategy (Refaeilzadeh et al. 2009), a particular case of *K*-fold cross-validation with *K* equal to *N*, the number of projects in the set. We trained models using the test cases of $N-1$ projects and used the test cases of the remaining project as the test set. The process was repeated *N* times to ensure each project would occur in the test set once.

## Research Method and Results

This section discusses the research methods employed to address the three main research questions targeted by our work.

### **RQ**_1_ - In Search of Suitable Metrics for Machine Learning-Based Test Smell Detection

*Research Method* As explained in Section 4, we focused on the metrics used by previous researchers when detecting test smells, i.e., we investigated whether a machine learning solution was suitable to combine structural and textual metrics considered in isolation by previous work. Table 4 lists and describes each considered test smell and the independent variables taken into account for each smell under investigation. These metrics captured the smelliness of tests under different perspectives, considering the size of fixtures and test suites, cohesion and coupling aspects of tests, and conceptual relationships between the methods composing test suites. We quantified the predictive power of each metric by computing their *information gain* (Quinlan 1986). This step was used as a *probing* method, i.e., this step allowed us to estimate the contribution provided by each metric. In addition, information gain has also been used as a feature selection instrument for **RQ**_2_, and **RQ**_3_: we indeed used as predictors the metrics having an information gain higher than zero, i.e., we discarded the metrics that did not provide any expected beneficial effect on the performance. The output of the information gain algorithm consists of a ranked list where the features of the model are placed in a descending manner, meaning that those contributing the most are placed at the top. We employed the *Gain Ratio Feature Evaluation* algorithm (Quinlan 1986) available in the Scikit-Learn library (Kramer 2016).

**Table 5:** The mean of the *information gain* obtained by all the considered metrics during the within- and cross-project validation

Test smell	Metric	Within-project	Cross-project
Eager test	NMC: Number of Method Calls	0.037	0.007
	TMC: Test Method Cohesion, i.e., the average textual similarity between all the pairs methods called by a test method	0.428	0.559
	TS: The extent to which the text within the method body is conceptually scattered	0.428	0.559
Mystery guest	NRF: Number of References to Files	0.661	0.042
	NRDB: Number of References to Database	0.015	0.001
Resource optimism	ERNC: state of External Resources, which are not files, Not Checked	0.012	0.007
	FRNC: state of File Resources Not Checked	0.052	0.022
Test redundancy	PR: Pair Redundancy, i.e., the ratio between the items covered by a test and those covered by another one	0.001	0.000
	SR: Suite, i.e., Redundancy the ratio between the items covered by a test compared and those covered by all other tests in the test suite	0.001	0.001

*Analysis of the Results* Table 5 reports the results for **RQ**_1_, considering the within- and cross-project scenarios. As for the *Eager Test* smell, we could notice that TMC and TS provide a higher information gain than NMC. Both these metrics are textual, and, according to our results, textual metrics seem to behave better than structural ones, possibly confirming the findings by Palomba et al. (2018). This result holds for both (of the) validation scenarios considered in our work. Perhaps more interestingly, it is worth discussing the low contribution of NMC. While an *Eager Test* is a test exercising multiple production methods, our results report that the number of method calls done by the test—which might be a proxy of the number of production methods exercised—is not a suitable metric. This result contradicts previous findings, raising questions on the metrics that may be used to identify *Eager Test* smells.

As for *Mystery Guest*, the number of references to files was the most impactful metric, especially in the within-project scenario. At the same time, the NRDB was found to be less impactful. Also in this case, the results were consistent in both validation scenarios. In any case, conceptually speaking, both the metrics were very close to the definition of the smell, hence possibly contributing to its detection. Likely, most considered systems store data using files, influencing our results.

When it turns to *Resource Optimism*, the information gain achieved for both (of) the considered metrics, i.e., ERNC and FRNC, is relatively less significant in both scenarios. This result is somehow surprising, as these metrics align with the definition of the smell—yet they are not only based on external files, possibly neglecting other data storage solutions. Our findings can suggest that further points of view, and therefore metrics, may be relevant.

Finally, when considering *Test Redundancy*, we found that the metrics had a very low information gain in both validation scenarios. On the one hand, this finding might be due to the limited diffuseness of this smell, i.e., we could find just 40 instances of this smell over 66 projects. On the other hand, the metrics considered were likely unable to characterize the problem, possibly making this smell detection hard.

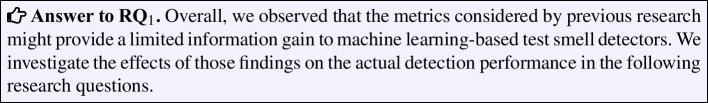



### **RQ**_2_ - Assessing the Performance of our Machine Learning-Based Test Smell Detector

*Research Method* When assessing the performance of the machine-learning models, we proceeded with a stepwise analysis of the various components included in the experimentation. We performed an *ablation* study to analyze the contribution of each configuration and training step to the overall models’ performance. We experimented with multiple combinations, i.e., we analyzed how the performance varies when including (and not) the feature selection step, the data balancing, and the hyper-parameter optimization, other than considering the performance variations given by the different validation procedures. In this way, we could also assess the best possible pipeline for the problem of test smell detection. To evaluate the performance of the various combinations experimented and address **RQ**_2_, we computed several state-of-the-art metrics such as *precision*, *recall*, *F-Measure* (Baeza-Yates et al. 2011), *Matthews Correlation Coefficient* (*MCC*) (Baldi et al. 2000), and the *Area Under the Curve - Precision-Recall (AUC-PR)*.

We statistically verified our conclusions by using the Friedman (Sheldon et al. 1996) and Nemenyi tests (Nemenyi 1963) on the distribution of MCC values of machine learning models over the different projects, configurations, and test smell types for statistical significance. We used the former to determine whether or not there is a statistically significant difference between the MCC value, while we used the latter to report its results using MCM (i.e., Multiple Comparisons with the best) plots (McMinn 2004). We used 0.05 as a significance level, so the elements plotted above the gray band were statistically larger than the others. In addition, the dots in the plot represented the median MCC that the algorithms obtained in the projects: a blue dot indicated that the MCC of an algorithm was statistically better than the other algorithms. In contrast, red dots indicated that the performance was not statistically different. To perform this last step, we relied on the nemenyi function available in R toolkit.^4^

*Analysis of the Results* Our study analyzed the machine learning approach when considering both within- and cross-project scenarios. For the sake of readability, we first discuss the results obtained from the ablation study conducted on the features, as the results of this step informed all the other steps. Afterward, we split the analysis of the results by validation strategy.

*Ablation Study for Feature Selection* To conduct such an ablation study, we relied on the outcome of the information gain analysis to understand whether and which features should have been excluded. The results of **RQ**_1_ reported that all the metrics considered provided a *non-null* information gain, indicating that none of them could be excluded by the set of features used by a machine learning instrument. Consequently, we could conclude that the ablation study on feature selection did not reveal findings that should have informed the set of features to use when building the machine learning-based detector.

**Fig. 1:**
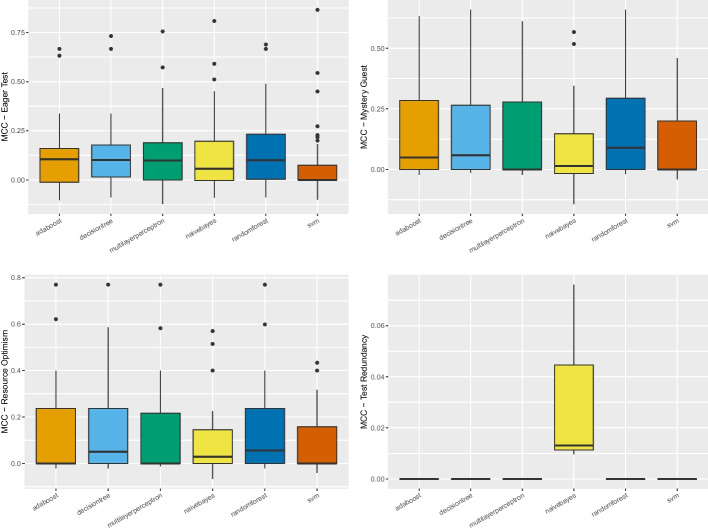
Boxplot representing the MCC values obtained by classifiers for all considered test smells in the within-project setting

These observations were also backed up by an additional analysis we performed when considering the performance of the machine learning-based detectors when relying on individual metrics as features. In the within-project scenario, we built nine machine learning-based detectors for each software project considered in the study, summing up to 108 configurations for each test smells. In the cross-project scenario, we devised additional 54 configurations for each test smell, i.e., nine machine learning-based detectors for each execution of the *Leave-One-Out Cross-Validation*. While the detailed results of this additional analysis are reported in our online appendix (Pontillo et al. 2023), we found the machine learning-based detectors perform even worse when relying on individual metrics than the detectors relying on all metrics. On the one hand, this finding corroborated the results from **RQ**_1_: all the metrics provide some information gain, and, therefore, they should be considered together when training a machine learning instrument. On the other hand, this finding suggests that the metrics are orthogonal to each other, meaning that they do not operate in a conflicting fashion when classifying the smelliness of test cases. In conclusion of this first step, we could observe that the best configuration of features to use is the one that includes all the metrics, and for this reason, the next sections describe the results obtained by this configuration.

**Fig. 2:**
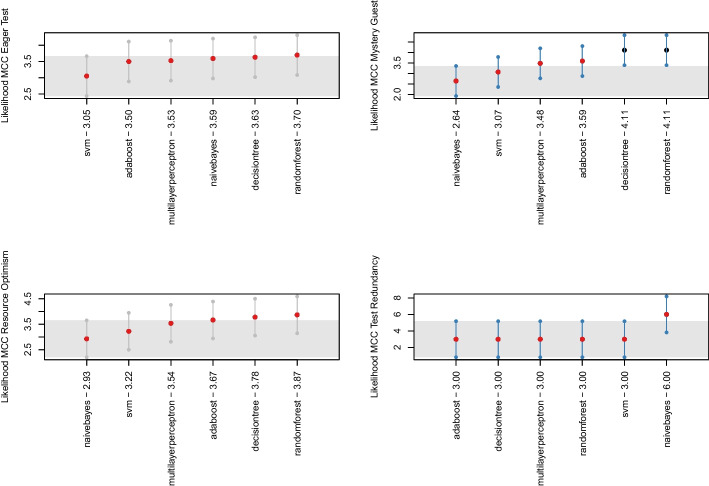
The likelihood of each model for the four test smells in within-project validation in Nemenyi rank in MCC. The circle dots are the median likelihood, while the error bars indicate the 95% confidence interval. 60% of likelihood means that a classification technique appears at the top rank for 60% of the studied projects

**Fig. 3:**
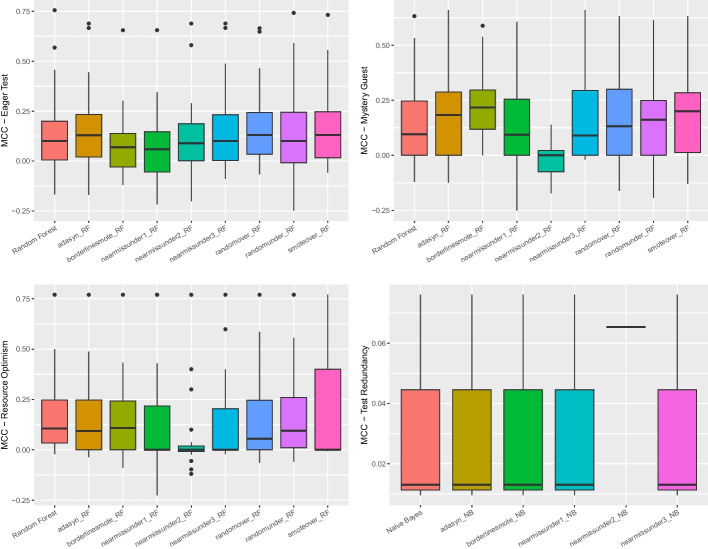
Boxplot representing the MCC values obtained by balancing techniques for all considered test smells in the within-project setting

*Within-project Results* The ablation study led us to build 108 configurations for each project—7,128 models in total. Each model was configured and run for each of the four test smells considered in our study, resulting in 28,512 different runs. We only discuss the best configuration for each test smell for readability while we report the full results in our online appendix (Pontillo et al. 2023).

Looking at Fig. 1, we could observe that the median MCC achieved by *Random Forest* on *Eager Test*, *Mystery Guest*, and *Resource Optimism* is slightly higher than the other algorithms (respectively 0.1, 0.09 and 0.05)—detailed result for all models are in our online appendix (Pontillo et al. 2023). In contrast, in the case of *Test Redundancy*, *Naive Bayes* seems to be the only classifier capable of detecting this smell the median was 0.01. The Friedman test showed that the distributions for *Eager Test* and *Resource Optimism* do not show statistically significant differences. However, we still decided to apply the Nemenyi test to all test smells to analyze which model showed higher values, even if not statistically significant. Figure 2 plots the outcome of the Nemenyi Test on the four test smells in the within-project validation. We can observe that for two test smells, i.e., *Eager Test* and *Resource Optimism*, no algorithm performed statistically better than others—all the dots are red. Differently, for *Mystery Guest*, *Random Forest* and *Decision Tree* are shown to achieve better performance than the others with a statistically significant difference. Based on these considerations, we will discuss the following results relying on *Random Forest* for *Eager Test*, *Resource Optimism*, and *Mystery Guest*. In contrast, *Naive Bayes* will be used for further analyses on *Test Redundancy*.

**Fig. 4:**
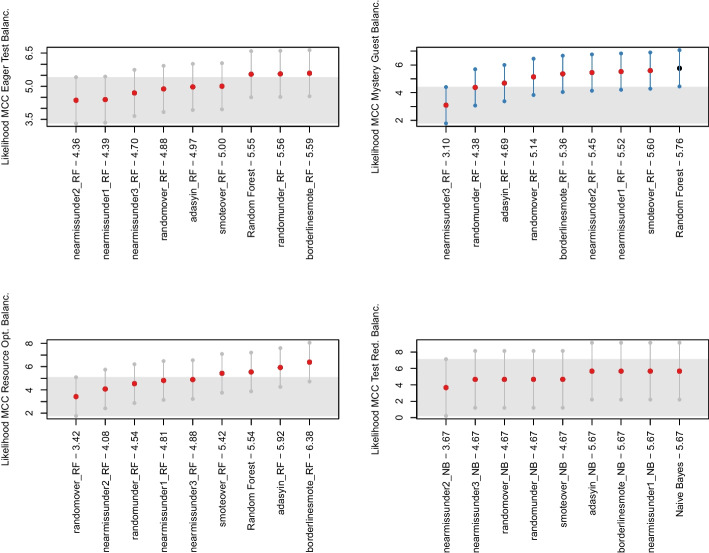
The likelihood of each balancing technique for the four test smells in within-project validation in Nemenyi rank in MCC. The circle dots are the median likelihood, while the error bars indicate the 95% confidence interval. 60% of likelihood means that a classification technique appears at the top rank for 60% of the studied projects

Concerning the impact of the balancing techniques, the Friedman test found no statistically significant differences between the distributions except for *Mystery Guest*. Figures 3 and 4 show the distributions of MCC and the Nemenyi ranks for each smell. We can observe that no balancing technique performed statistically better than the others in the cases of *Eager Test* and *Resource Optimism*—*BorderlineSMOTE* seems to have slightly higher performance. When it turns to *Mystery Guest*, the *Random Forest* classifier, without any balancing algorithm, performed statistically better than all the alternatives. This observation is also true for *Test Redundancy*, yet in this case, the performance differences are not statistically significant. All in all, our findings seem to corroborate previous observations showing that balancing algorithms are not always effective in the context of code smell detection (Pecorelli et al. 2019a).

Therefore, the best machine learning classifiers for the four test smells analyzed are (i) *Random Forest* with *Borderline-SMOTE* for *Eager Test*, (ii) *Random Forest* for *Mystery Guest*, (iii) *Random Forest* with *Borderline-SMOTE* for *Resource Optimism*, and (iv) *Naive Bayes* for *Test Redundancy*.

The last step of the ablation study concerns hyperparameter optimization. We compared the performance of the best models with and without hyperparameters optimization to understand whether and to what extent such additional steps could improve the models.

It is important to point out that, since we considered several systems, we needed to aggregate the results achieved for each system to have a more transparent overview of the performance (Antoniol et al. 2002). Therefore, we aggregated the obtained confusion matrices before computing Precision, Recall, F-Measure, and MCC. Moreover, we must also point out that we could not produce results for all the smells analyzed and all individual projects. By diagnosing the reasons behind the failures of the models, we identified a main factor: on some projects, the number of test smells was equal to one, causing a training error. Therefore, we created and tested machine learning models for 37 projects for *Eager Test*, 28 projects for *Mystery Guest*, 18 projects for *Resource Optimism*, and three projects for *Test Redundancy*.

**Table 6:** Aggregate results for *Precision*, *Recall*, *Accuracy*, *F-Measure*, *MCC*, and *AUC-PR* without (i.e., “w/o HT”) and with (i.e., “w/ HT”) the hyper-parameter optimization by Random Search in the within-project setting

	Precision		Recall		Accuracy	
Test smell	w/o HT	w/ HT	w/o HT	w/ HT	w/o HT	w/ HT
Eager test	0.47	0.48	0.53	0.54	0.68	0.68
Mystery guest	0.64	0.64	0.34	0.34	0.83	0.84
Resource opt.	0.33	0.33	0.31	0.36	0.85	0.84
Test red.	0.08	0.01	1.00	0.97	0.05	0.03
	F-Measure		MCC		AUC-PR	
Test smell	w/o HT	w/ HT	w/o HT	w/ HT	w/o HT	w/ HT
Eager test	0.50	0.51	0.27	0.28	0.49	0.50
Mystery guest	0.45	0.44	0.39	0.38	0.59	0.55
Resource opt.	0.32	0.34	0.24	0.25	0.53	0.53
Test red.	0.01	0.01	0.01	0.01	0.52	0.52

Table 6 shows the achieved performance in terms of *Precision*, *Recall*, *Accuracy*, *F-Measure*, *MCC*, and *AUC-PR*. The result immediately highlights that the performance of the approaches is generally low. The maximum F-Measure achieved is for *Eager Test* (i.e., 51%). Analyzing the MCC, we notice that the performance ranges from 0.01 (*Test Redundancy*) to 0.39 (*Mystery Guest*). Overall, we found that the hyper-parameter optimization did not improve the performance as much as to justify the high computational cost required.

**Fig. 5:**
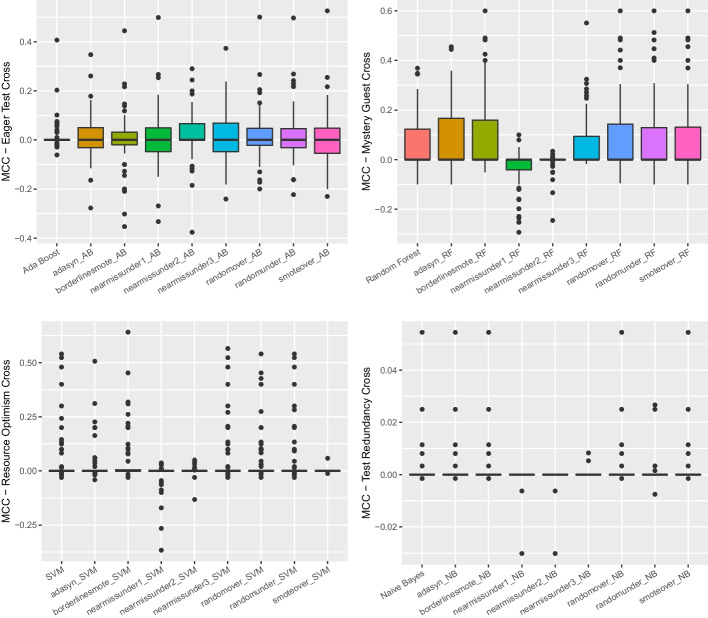
Boxplot representing the MCC values obtained by balancing techniques during the cross-project validation for all considered test smells

*Cross-project Results* Regarding the cross-project validation, we performed the same *ablation* study applied for the within-project validation. While the entire process is shown in our online appendix (Pontillo et al. 2023), here we only report and discuss the results of the Nemenyi Test and the distribution for each test smell after applying the various balancing techniques.

Differently from the within-project configuration, we found *Ada Boost* to be the best classifier for *Eager Test* and *Support Vector Machine* for *Resource Optimism* in the cross-project setting. As for *Mystery Guest* and *Test Redundancy*, the best classifiers are the same as the within-project setting, namely *Random Forest* and *Naive Bayes*, respectively.

**Fig. 6:**
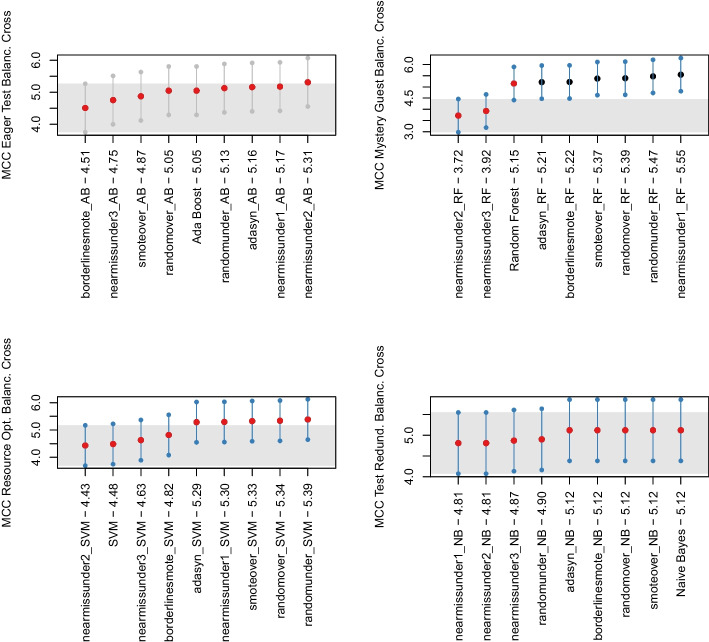
The likelihood of each balancing technique for the four test smells in cross-project validation in Nemenyi rank in MCC. The circle dots are the median likelihood, while the error bars indicate the 95% confidence interval. 60% of likelihood means that a classification technique appears at the top rank for 60% of the studied projects

Figure 5 reports the boxplots showing the performance of different data balancing algorithms in the cross-project setting. As we can observe, the performance is generally poor, with MCC values close to zero. However, differently from the within-project configuration, the Friedman test and the Nemenyi test found statistically significant differences between the experimented data balancing techniques. From Fig. 6, we can observe that the various distributions exhibit statistical significance except for *Eager Test*. In addition, *Mystery Guest* shows several blue dots, i.e., some balancing techniques perform statistically better than others. Our results report that, in the cross-project context, undersampling techniques are more useful than oversampling techniques except for *Test Redundancy*. In this case, the classifier without any balancing technique performed better, although no statistically-significant difference is reported compared to the other techniques. These differences with within-project validation can be explained by the training data containing way more instances in a cross-project setting, thus enabling more exhaustive training of the machine learning models.

Based on the results discussed so far, the following discussion will focus on (i) *Ada Boost* with *NearMiss2* for the *Eager Test* detection, (ii) *Random Forest* with *NearMiss1* for the *Mystery Guest* detection, (iii) *SVM* with *Random Undersampling* for the *Resource Optimism* detection, and (iv) *Naive Bayes* for the *Test Redundancy* detection.

Table 7 reports the aggregate results in terms of *Precision*, *Recall*, *Accuracy*, *F-Measure*, *MCC*, and *AUC-PR* of the best models with and without hyper-parameter optimization. The results obtained were generally low, even more than the within-project validation. The maximum F-Measure achieved was for *Mystery Guest* (40%), while the MCC ranges from -0.01 to 0.3. Hence, cross-project validation is ineffective in classifying negative class samples. Finally, analyzing the AUC-PR, the maximum results obtained was 46% for *Mystery Guest*. It is important to point out that also for the cross-project validation, the hyper-parameter optimization did not improve the performance.

**Table 7:** Aggregate results for *Precision*, *Recall*, *Accuracy*, *F-Measure*, *MCC*, and *AUC-PR* without (i.e., “w/o HT”) and with (i.e., “w/ HT”) the hyper-parameter optimization by Random Search in the cross-project setting

	Precision		Recall		Accuracy	
Test smell	w/o HT	w/ HT	w/o HT	w/ HT	w/o HT	w/ HT
Eager test	0.27	0.30	0.64	0.54	0.42	0.53
Mystery guest	0.44	0.44	0.37	0.37	0.82	0.82
Resource opt.	0.25	0.24	0.32	0.30	0.87	0.87
Test red.	0.004	0.01	0.97	0.97	0.05	0.03
	F-Measure		MCC		AUC-PR	
Eager test	0.38	0.39	-0.01	0.06	0.32	0.33
Mystery guest	0.40	0.40	0.30	0.30	0.46	0.41
Resource opt.	0.28	0.26	0.22	0.20	0.27	0.28
Test red.	0.01	0.01	0.01	0.00	0.41	0.13

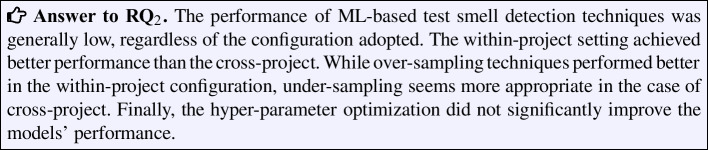



### **RQ**_3_ - Comparing Machine Learning- and Heuristic-Based Techniques for Test Smell Detection

*Research Method* While the results achieved in the context of **RQ**_2_ reported that machine learning-based test smell detectors did not sufficiently perform, we still conducted a benchmark study to address two specific objectives. On the one hand, we could assess the real usefulness of the machine learning-based technique: should our model be less performing than the baselines, its practical use would be further limited, and, because of that, we could recommend researchers invest effort in the improvement of heuristic approaches rather than of machine learning-based solutions. On the other hand, we could measure the extent to which our technique compares to existing approaches, thus understanding the strengths and weaknesses of the proposed test smell detector compared to existing detectors. More particularly, our study aimed at comparing the machine learning-based test smell detectors against the three heuristic-based baselines described in the following:

tsDetect (Peruma et al. 2020) We selected this tool as it represents the current state of the art in test smell detection (Aljedaani et al. 2021) and can detect the highest number of test smell types. Out of the four test smells included in our study, tsDetect could identify three of them, i.e., *Eager Test*, *Mystery Guest*, and *Resource Optimism*. In particular, the first is detected by computing the number of the multiple calls made by a test method to multiple production methods. The second is identified by analyzing whether a test method contains instances of files and database classes. Finally, the third is identified by looking at whether a test method utilizes a File instance without calling the method exists(), isFile(), or notExist().

**Fig. 7:**
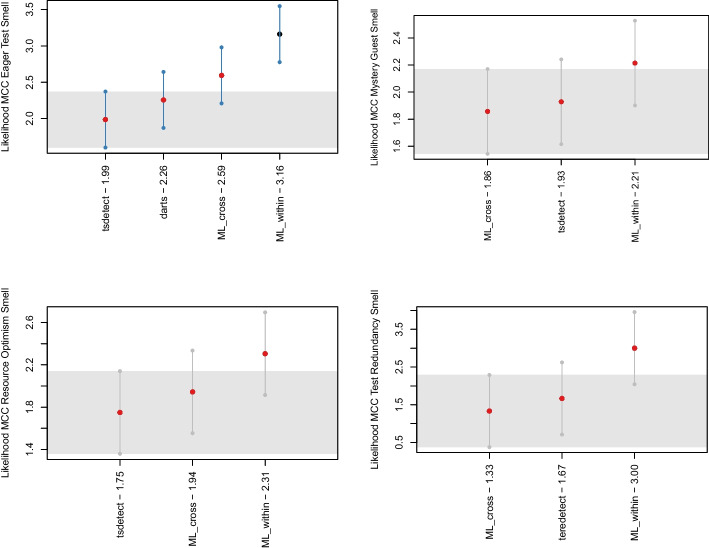
The likelihood of the heuristic- and machine learning-based techniques to detect the four test smells ranked by Nemenyi computed on the MCC. The circle dots are the median likelihood, while the error bars indicate the 95% confidence interval. 60% of likelihood means that a classification technique appears at the top rank for 60% of the studied projects

TeReDetect (Koochakzadeh and Garousi 2010) We selected this tool as it is the only one to detect *Test Redundancy* smell instances. The tool detects the smell by computing code coverage and analyzing whether two tests cover similar paths.

Darts (Lambiase et al. 2020) The model built for *Eager Test* relies on an information retrieval metric (i.e., TC). For this reason, we also considered it worth comparing the model against an information retrieval-based heuristic technique, which is the one implemented within Darts (Lambiase et al. 2020). The tool relies on the detection rule proposed by Palomba et al. (2018). It detects *Eager Test* instances through a two-step process: first, the test method calls are replaced with the actual production code methods called by the test method; then, the conceptual cohesion metric is computed, taking into account the constituent methods and, whether this metric exceeds 0.5 the smell is detected.

We run the heuristic approaches against the same systems considered in **RQ**_2_ to enable a fair comparison. None of these heuristic tools required additional configuration, i.e., they could be run against the source code without the need to specify any parameter: this ensured the execution of their original implementations, hence avoiding possible bias due to the wrong configuration of the tools. We employed the same evaluation metrics used to assess the machine learning models, i.e., *Precision*, *Recall*, *F-Measure*, and *MCC*. Similarly to **RQ**_2_, we also statistically verified the validity of the findings between our machine learning-based detector and baseline techniques using the Nemenyi test (Nemenyi 1963) on the distribution of MCC values they obtained.

**Table 8:** Aggregate results for *Precision*, *Recall*, *F-Measure*, and *MCC*, comparing the machine learning approach to TsDetect

	Precision		Recall	
Test smell	ML within	TSDETECT	ML within	TSDETECT
Eager test	0.47	0.37	0.53	0.17
Mystery guest	0.64	0.42	0.34	0.44
Resource opt.	0.33	0.21	0.31	0.37
	F-measure		MCC	
Test smell	ML within	TSDETECT	ML within	TSDETECT
Eager test	0.50	0.23	0.27	0.06
Mystery guest	0.45	0.43	0.39	0.29
Resource opt.	0.32	0.27	0.24	0.15
	Precision		Recall	
Test smell	ML cross	TSDETECT	ML cross	TSDETECT
Eager test	0.27	0.35	0.64	0.16
Mystery guest	0.44	0.40	0.37	0.40
Resource opt.	0.25	0.18	0.32	0.37
	F-measure		MCC	
Test smell	ML cross	TSDETECT	ML cross	TSDETECT
Eager test	0.38	0.22	-0.01	0.06
Mystery guest	0.40	0.40	0.30	0.29
Resource opt.	0.28	0.25	0.22	0.17

**Table 9:** Aggregate results for *Precision*, *Recall*, *F-Measure*, and *MCC*, comparing the machine learning approach to Darts

	Precision		Recall	
Test smell	ML within	Darts	ML within	Darts
Eager test	0.47	0.33	0.53	0.31
	F-Measure		MCC	
Test smell	ML within	Darts	ML within	Darts
Eager test	0.50	0.32	0.27	0.04
	Precision		Recall	
Test smell	ML cross	Darts	ML cross	Darts
Eager test	0.27	0.30	0.64	0.31
	F-measure		MCC	
Test smell	ML cross	Darts	ML cross	Darts
Eager test	0.38	0.30	-0.01	0.03

*Analysis of the Results* Similarly to **RQ**_2_, we split the analysis of the results by validation strategy so that we could benchmark the machine learning approach and heuristic-based techniques in two different scenarios. *Within-project Results.* Figure 7 reports the outcome obtained from the Nemenyi test comparing the various distributions of MCC. We can observe that for *Eager Test*, the machine learning in a within-project setting performs statistically better compared to the other approaches. For *Mystery Guest*, *Resource Optimism*, and *Test Redundancy*, the machine learning approach has higher performance even if there are no statistically significant differences.

**Table 10:** Aggregate results for *Precision*, *Recall*, *F-Measure*, and *MCC*, comparing the machine learning approach to TeReDetect

	Precision		Recall	
Test smell	ML within	TeReDetect	ML within	TeReDetect
Test red.	0.01	0.00	1.00	0.00
	F-Measure		MCC	
Test smell	ML within	TeReDetect	ML within	TeReDetect
Test red.	0.01	0.00	0.01	−0.01
	Precision		Recall	
Test smell	ML cross	TeReDetect	ML cross	TeReDetect
Test red.	0.01	0.00	0.97	0.00
	F-Measure		MCC	
Test smell	ML cross	TeReDetect	ML cross	TeReDetect
Test red.	0.01	0.00	0.01	-0.01

**Table 11:** The overlap results in a within-project scenario. We reported the results for each test smell by comparing the machine learning-based approach to the heuristic-based one

Eager test		
ML _corr_ $\cap$ Darts_corr_	ML _corr_ $\setminus$ Darts_corr_	Darts _corr_ $\setminus$ ML _corr_
26%	53%	21%
ML _corr_ $\cap$ TsDetect_corr_	ML _corr_ $\setminus$ TsDetect_corr_	TsDetect _corr_ $\setminus$ ML _corr_
12%	76%	12%
Mystery guest		
ML _corr_ $\cap$ TsDetect_corr_	ML _corr_ $\setminus$ TsDetect_corr_	TsDetect _corr_ $\setminus$ ML _corr_
72%	5%	23%
Resource optimism		
ML _corr_ $\cap$ TsDetect_corr_	ML _corr_ $\setminus$ TsDetect_corr_	TsDetect _corr_ $\setminus$ ML _corr_
60%	13%	27%
Test redundancy		
ML _corr_ $\cap$ TeReDetect_corr_	ML _corr_ $\setminus$ TeReDetect_corr_	TeReDetect _corr_ $\setminus$ ML _corr_
0%	100%	0%

Tables 8 and 9 show the aggregate results for *Eager Test*, *Mystery Guest*, and *Resource Optimism* over the machine learning approach and two heuristic-based techniques (i.e., TsDetect and Darts). Concerning the three test smells detected by TsDetect, the performance was generally lower in terms of *Precision*, *F-Measure*, and *MCC* compared to machine learning-based approaches. Looking at the *Recall*, we notice that compared with the machine learning approach, TsDetect has higher values when it comes to the detection of *Mystery Guest* and *Resource Optimism*.

The results obtained by Darts confirmed that the machine learning-based approach performed better than the heuristic baselines for all the metrics evaluated, e.g., the MCC (27% vs. 4%).

To further elaborate on the differences between the approaches we conducted an additional analysis focused on understanding the overlap among them. Given two prediction models m_i_ and m_j_, we computed (i) the number of test smells correctly predicted by both m_i_ and m_j_ and (ii) the number of test smells correctly predicted by only m_i_ and missed by m_j_.

The overlap analysis for the within-project scenario is reported in Table 11. The analysis confirms the previous results and shows that the machine learning-based approach detects more test smells than the heuristic-based approaches when analyzing *Eager Test* and *Test Redundancy*. For *Mystery Guest* and *Resource Optimism*, the amount of common predictions is higher than that of the individual machine learning- and heuristic-based approaches.

*Cross-Project Results* Different conclusions can be drawn in the cross-project setting. While the cross-project machine learning is still shown to perform better than TsDetect in terms of MCC for most of the code smells under analysis, there is no statistical significance. Moreover, looking at the other indicators, we notice that the machine learning approach is, overall, less precise. The explanation behind this result could be that in a cross-project configuration, instances coming from heterogeneous systems are used for training. Therefore, the classifiers are brought to infer a more generic detection and generate more false positives.

The only smell that deserves a separate discussion is *Test Redundancy*, whose results are reported in Table 10. In this case, the performance of the various approaches is very low (close to zero), even if machine learning still performs slightly better than TeReDetect, particularly for the Recall.

**Table 12:** The overlap results in a cross-project scenario. We reported the results for each test smell by comparing the machine learning-based approach to the heuristic-based one

Eager test		
ML _corr_ $\cap$ Darts_corr_	ML _corr_ $\setminus$ Darts_corr_	Darts _corr_ $\setminus$ ML _corr_
26%	60%	14%
ML _corr_ $\cap$ TsDetect_corr_	ML _corr_ $\setminus$ TsDetect_corr_	TsDetect _corr_ $\setminus$ ML _corr_
15%	78%	7%
Mystery guest		
ML _corr_ $\cap$ TsDetect_corr_	ML _corr_ $\setminus$ TsDetect_corr_	TsDetect _corr_ $\setminus$ ML _corr_
14%	67%	19%
Resource optimism		
ML _corr_ $\cap$ TsDetect_corr_	ML _corr_ $\setminus$ TsDetect_corr_	TsDetect _corr_ $\setminus$ ML _corr_
11%	71%	18%
Test redundancy		
ML _corr_ $\cap$ TeReDetect_corr_	ML _corr_ $\setminus$ TeReDetect_corr_	TeReDetect _corr_ $\setminus$ ML _corr_
0%	100%	0%

Looking at the overlap analysis for the cross-project scenario (reported in Table 12), the results showed that the machine learning-based approach detects more test smells than the heuristic-based approaches for all test smells. In addition, we could observe that for *Eager Test*, only 26% of the predicted smells are in common between machine learning and Darts and further decreased to 15% when analyzing TsDetect. A similar discussion can be drawn for *Mystery Guest* and *Resource Optimism*, while again for *Test Redundancy*, this analysis is infeasible because the number of smells detected by TereDetect but missed by the machine learning approach is zero (Table12).

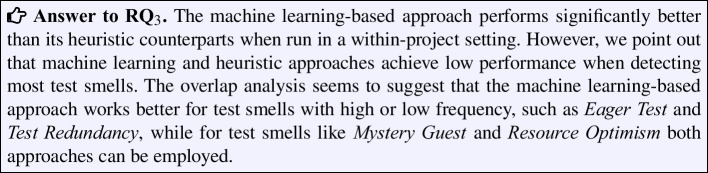



## Discussion, Further Analysis, and Qualitative Insights

Our findings reveal several points worthy of further analysis and discussion, which we elaborate on in this section.

### Machine Learning-based Test Smell Detection: How Bad Is It?

According to our findings, a machine learning-based detector might perform better than heuristic-based alternatives. Yet, this seems not to be enough, as a key result of our investigation concerns the low performance achieved by the machine learning-based detector in terms of all the evaluation metrics considered. Regardless of the type of test smell considered and the machine learning configuration adopted in within- and cross-project scenarios, it cannot solve the problem effectively. To provide a more pragmatic measure of how low the performance achieved is, we conducted an additional analysis to compare the machine learning-based detector with the so-called *dummy* classifiers, i.e., classifiers that make predictions ignoring the input features. More specifically, we compared the best model, both in within- and cross-project scenarios, coming from our empirical study with three baselines such as (i) the *Optimistic Constant Classifier*, which consistently classifies an instance as smelly; (ii) the *Pessimistic Constant Classifier*, which consistently classifies an instance as non-smelly; and (iii) a *Random Classifier*, which randomly classifies an instance as smelly or non-smelly. Through this comparison, we could assess how far we are, as researchers, to the definition of a usable and effective machine learning-based test smell detector by measuring the distance between the performance it achieved and those of simple classifiers. In addition, if any of these baselines would have outperformed our solution, this might have potentially indicated an issue with the features exploited by the model, i.e., if a classifier that ignores the input features perform better than one based on features, this would imply that the features themselves are not impactful enough.

Following the research method taken in previous studies (Haiduc et al. 2012), we compared Type I and Type II errors, namely the total number of false positive and false negative errors, respectively.

**Table 13:** Comparison between the experimented machine learning-based test smell detector and the dummy classifiers in the within-project scenario

	ML-based approach		Optimistic constant	
Test smell	Type I	Type II	Type I	Type II
Eager test	1,524 (17%)	1,240 (14%)	6,079 (70%)	0 (0%)
Mystery guest	239 (3%)	817 (10%)	6,118 (80%)	0 (0%)
Resource opt.	445 (7%)	481 (8%)	5,576 (89%)	0 (0%)
Test red.	2,302 (72%)	0 (0%)	3,169 (99%)	0 (0%)
	Pessimistic constant		Random constant	
Test smell	Type I	Type II	Type I	Type II
Eager test	0 (0%)	2,648 (30%)	3,246 (37%)	1,293 (15%)
Mystery guest	0 (0%)	1,487 (20%)	3,409 (45%)	764 (10%)
Resource opt.	0 (0%)	688 (11%)	2,995 (48%)	346 (6%)
Test red.	0 (0%)	40 (1%)	2,089 (65%)	26 (1%)

Table 13 reports the results obtained when considering the within-project scenario. As for the Type I errors, the machine learning-based approach outperformed both the *Optimistic Constant Classifier* and *Random Classifier*, reaching lower false positive rates for all the test smells. For instance, the false positive rate for *Eager Test* was 17%, namely 53% and 20% lower than the *Optimistic Constant Classifier* and *Random Classifier*, respectively. At the same time, the difference compared to the *Pessimistic Constant Classifier* was still evident, especially when considering the absolute number of errors, particularly in the case of *Test Redundancy*, where the machine learning-based approach output 2,302 false positives (72%). Based on these results, we could conclude that the machine learning approach was too eager to recommend the smelliness of test cases, producing a notable amount of false positives.

When it turns to Type II errors, we could observe that the approach obtained results close to those of the *Random Classifier* for all the considered test smells, with absolute numbers indicating a similar behavior. By interpreting those numbers, we could conclude that the machine learning-based approach was often unable to properly recognize the smelliness of test code, performing no better than a random choice. This finding is even more worrisome than the one obtained for the Type I errors, as it possibly indicates that the features or the configuration exploited by the approach often could not correctly characterize the presence of test smells.

The conclusions drawn were similar when considering the cross-project scenario. As shown in Table 14, the trend looks similar to what was just discussed. Regarding Type I error, the machine learning-based approach typically worked better than the *Optimistic Constant Classifier* and *Random Classifier* alternatives. The only exception concerned with *Eager Test*: in the cross-project scenario, the false positive rate was indeed higher than the previous case when compared to the *Random Classifier*, possibly indicating that the approach was even more prone to highlight the presence of test smells. As for the Type II errors, we could instead confirm the similar behavior between the machine learning-based approach and the *Random Classifier*.

On the basis of the argumentation above, we can conclude that the machine learning-based detector was quite unstable, both considering false positives and negatives: this suggests that either the features or other characteristics of the problem exploited were not suitable enough for the classification task - we elaborate on this matter in the next section. Also, our results suggest that the research on machine learning-based test smell detection is still far from reaching a decent point. Indeed, the current solution is unsuitable for a practical case and too close to dummy alternatives. On the one hand, in the within-project scenario, a model ensemble (i.e., *Random Forest*) is the best classifier for three out of four test smells. On the other hand, in the cross-project scenario, two ensemble models (ii.e., *Random Forest* and *AdaBoost*) are the best classifiers for two out of four test smells. Please consider that Random Forest is an ensemble of pruned decision trees, where each decision tree is built using Bootstrap Aggregating (i.e., Bagging), and the combination of the prediction of the decision trees is performed by using majority voting. Hence, the results of RQ2 suggest that ensemble learning can help achieve better performance, and their employment can leverage the results obtained by other classifiers (e.g., *Naive Bayes*, *Multi-layer Perceptron*, and *Support Vector Machine*). In this sense, our work may pose the basis for additional studies, for instance, by targeting a larger variety of machine learning and natural language processing techniques, which might potentially improve the test smell detection capabilities by relying on different data representations and/or features.

**Table 14:** Comparison between the experimented machine learning-based test smell detector and the dummy classifiers in the cross-project scenario

	ML-based approach		Optimistic constant	
Test smell	Type I	Type II	Type I	Type II
Eager test	4,578 (48%)	942 (10%)	6,934 (72%)	0 (0%)
Mystery guest	723 (8%)	955 (10%)	8,099 (84%)	0 (0%)
Resource opt.	691 (7%)	492 (5%)	8,903 (92%)	0 (0%)
Test red.	9,105 (95%)	1 (0.01%)	9,593 (99%)	0 (0%)
	Pessimistic constant		Random constant	
Test smell	Type I	Type II	Type I	Type II
Eager test	0 (0%)	2,699 (28%)	3,485 (36%)	1,388 (14%)
Mystery guest	0 (0%)	1,534 (16%)	4,121 (43%)	780 (8%)
Resource opt.	0 (0%)	730 (8%)	4,462 (46%)	364 (4%)
Test red.	0 (0%)	40 (0.4%)	4,822 (50%)	27 (0.3%)

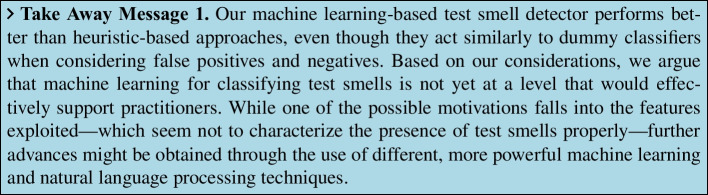



### Test Smell Detection: A Research Field to Revisit?

The underwhelming performance demonstrated by the machine learning-based approach and the limitations exposed through the comparison with the dummy classifiers raises significant concerns regarding the current approach to test smell detection. The analysis of false positive and negative rates suggests that multiple aspects should be revisited in terms of either features or formulation of the test smell detection problem. In the first place, the probing and ablation studies conducted on the features (**RQ**_1_ and **RQ**_2_) highlighted that, despite they all contribute to increase the prediction power, their actual contribution is limited and, indeed, the resulting performance improved when putting them together, as the machine learning-based approach could exploit the orthogonality between the features. Perhaps more importantly, the results for **RQ**_3_ suggest that the performance of the heuristic-based approaches is slightly lower, possibly highlighting fundamental, general problems pertaining to all test smell detectors. Indeed, when experimenting with those heuristic-based approaches against a large dataset of manually-validated instances, we were unable to generalize the performance reported in the original papers (Peruma et al. 2020; Koochakzadeh and Garousi 2010; Lambiase et al. 2020). These observations call for some more reflections. To further understand those aspects and provide the research community with insights into the challenges that should be addressed in future research, we proceeded with an additional qualitative investigation into the false positive and false negative instances output by machine learning- and heuristic-based approaches.

Our goal was to identify and classify the root causes of failure for each considered test smell so that we could point out indications for designing more accurate test smell detectors. To this aim, we set up a similar inspection process as described in Section 4. This time, the first and third authors of the paper took the role of inspectors. They manually went through the erroneous instances predicted by the experimented approaches, attempting to elicit the potential motivation(s) behind the errors. The inspectors first individually analyzed all the false positive and negative instances, writing down notes and observations to be further discussed—this task took around 100 hours/person. Afterward, they opened a discussion to elaborate on their individual observations: this was implemented through a Skype meeting that took around two hours. The outcome was a collection of representative qualitative examples that could explain the reasons behind the failures of the machine learning-based approach. Such a collection was finally discussed with the other paper authors, who provided additional feedback. In the following, we report on the specific root-cause analysis performed for each test smell, although there is a general consideration to make. From our additional analysis, we could realize that the errors made by the experimented detectors were similar, as these errors come from inaccurate interpretation of the test smell sources, improper measurement of the characteristics of those smells, or inappropriate treatment of corner cases. In other terms, the causes of failure are the same for all the detectors and may provide insight to improve the design of such detectors.

*Eager Test* When considering this test smell, we could classify three main root causes leading the approaches to fail. More specifically:

*1. Misleading definition of the problem* First and foremost, we identified 458 test cases with a serious concern regarding the definition of *Eager Test* enclosed by the detectors. Van Deursen et al. defined this test smell as a *“test method [that] checks several methods of the object to be tested”* (Van Deursen et al. 2001). Consequently, the structural detector identifies the smell by considering the number of production method calls, while the textual detector computes the conceptual similarity between the methods exercised by the test. The machine learning-based approach combines these metrics. The problem with the definition arises because it does not explicitly consider the difference between *intra-method* and *intra-class* unit testing (Pezzè and Young 2008). In particular, when designing unit test cases, two levels of granularity should be preserved (Harrold et al. 1992; Pezzè and Young 2008; Orso and Silva 1998). On the one hand, developers should create tests covering *individual* methods of the production code, i.e., *intra-method* (Pezzè and Young 2008) or *basic-unit testing* (Orso and Silva 1998). On the other hand, they should implement tests exercising the interaction between the methods of the class to verify additional execution paths of the production code that would not be covered otherwise, i.e., *intra-class* (Pezzè and Young 2008) or *unit testing* (Orso and Silva 1998). While it is reasonable to consider smelly an intra-method test that exercises more production methods, it is not the same for intra-class tests: these must necessarily call more production methods to perform unit testing effectively and should not be considered smelly. Unfortunately, the definition provided by Van Deursen et al. (2001) does not account for unit test granularity, possibly biasing the interpretation of the smell to enclose within the detectors. As a consequence, the vast majority of false positive instances were due to the presence of intra-class tests that were erroneously classified as *Eager Test*, but that instead should not be considered as such. A representative example is shown in Listing 1.

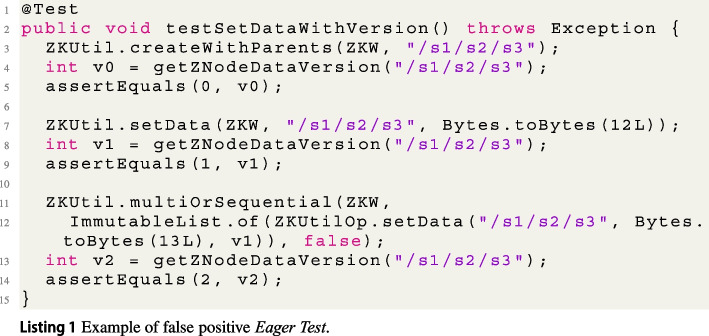



The test exercises a class named ZKUtil of the HBase project, i.e., a framework implementing a centralized service to maintain configuration information and provide distributed synchronization. The production method under test is named setData and is responsible for storing version data within an internal data structure. The test exercises an individual production method, i.e., setData, yet it calls various methods of the same production class, i.e., createWithParents and multiOrSequential. All the experimented detectors classified this instance as smelly. However, this is a false positive case because the calls performed to the production class methods are required to experiment with the setData method with different configurations to cover an execution path that could not be covered without performing those calls. For this reason, the test cannot be considered an *Eager Test*. Based on the argumentations above, we argue that the definition of this smell should be revisited to consider the levels of granularity that should be preserved in unit testing.

*2. Inability to Handle Mocks* When writing unit test cases, developers may simulate dependencies’ expected behaviors through the use of mock objects (Mackinnon et al. 2000). According to our analysis, in 380 test cases, the use of mocks represents a second threat to the accuracy of the detectors. In particular, when simulating the behavior of the dependencies, developers have to add a call to a mock object. This addition should not influence the test smell detectors, yet it does. In other terms, the metrics employed by the detectors do not consider mocking practices. Listing 2 presents an example.

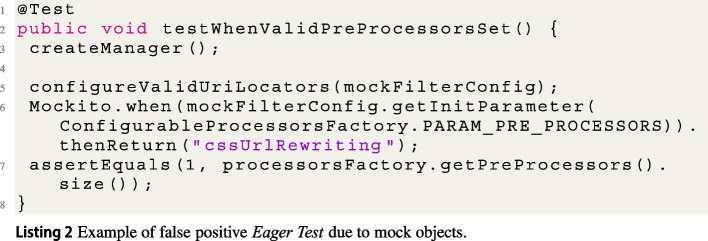



As shown, the test testWhenValidPreProcessorsSet leverages the *Mockito* framework,^5^ a well-known instrument to enable mocking, to simulate the behavior of the ConfigurableProcessorsFactory class and get parameters to use within the test. In this case, all detectors failed, as they mistakenly accounted for this call. As such, the definition of mocking-aware metrics would boost test smell detection capabilities.

*3. Limited Information Gathering* The third issue identified in 1,738 cases, significantly impacted the amount of false negative instances of all the experimented detectors. The limited information gathering arises when a detector has no or limited access to the production class related to the test method under account. More specifically, the metrics exploited to characterize *Eager Test* instances assume the existence of a linking between the test method under consideration and its corresponding production class., e.g., this linking is required to estimate the amount of calls made by the test method to the production class or compute the textual similarity metrics between the production methods involved in the test case. Unfortunately, such a linking is not always available nor reliable. All the experimented detectors perform an initial information-gathering phase which consists of linking test classes and methods to production code through a traceability technique based on pattern matching and naming conventions. In particular, this traceability technique takes the name of test class as input (e.g., DoubleConverterTest.java) and looks for the production class having the same name of the test class after removing the suffix or prefix Test (e.g., DoubleConverter.java). In case the search succeeds, the test class is associated to the production class and, in a subsequent information-gathering phase, the individual test methods of the test suite are linked to production methods using the same traceability technique. In the case the search fails, the linking is not performed and, therefore, the *Eager Test* detection fails. In this respect, there are two considerations to make. In the first place, the traceability technique employed by the tools is well-known in literature and has been experimented multiple times (Qusef et al. 2014; Van Rompaey and Demeyer 2009; Parizi et al. 2014), showing an accuracy close to 85%, which is comparable with more sophisticated but less scalable techniques (e.g., the slicing-based approach proposed by Qusef et al. (2014)). Of course, the overall accuracy of the test smell detection process is bounded to the accuracy of the linking process. As such, the improvements in the field of traceability recovery might provide insights into the field of test smell detection. In the second place, it is also worth discussing the sneakiest failure motivation, where the linking is correctly performed but the information available in the production class is not sufficient to perform the detection. To reason on this motivation, let us consider the example shown in Listing 3.

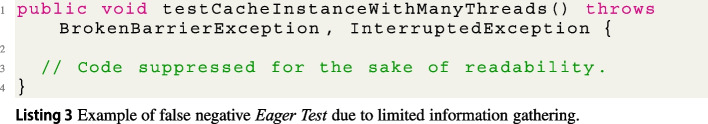



The test method belongs to the test suite EmbeddedJSPResultTest and has been classified as an *Eager Test* instance. According to the outcome of the information gathering phase, the test suite was linked to the EmbeddedJSPResult production class. Nonetheless, such a production class was only an interface for another class, i.e., JSPRuntime, which was responsible for the actual operations exercised by the testCacheInstanceWithManyThreads method. More specifically, the code of the EmbeddedJSPResult class is shown in Listing 4.

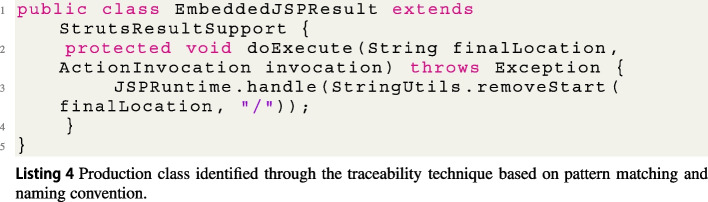



As shown in the listing, EmbeddedJSPResult just contains one method, i.e., doExecute, that delegates its own operations to the method handle of the JSPRuntime class. Because of that, EmbeddedJSPResult does not contain any method that could be linked to the testCacheInstanceWithManyThreads test and, for this reason, the test smell detectors could not compute the metrics that would have allowed its detection. In other terms, we may consider this example as a case of *conceptual false positive* link given by the traceability technique, i.e., the link is technically correct, yet the linked class is not the actual production class under test. On the one hand, the use of more advanced test-to-code traceability techniques (e.g., Qusef et al. 2014; Parizi et al. 2014) might boost the overall test smell detection capabilities. On the other hand, the example provided may inform the possible improvements to make in terms of test-to-code traceability based on pattern matching and naming convention. As a final point of discussion, we may argue that the EmbeddedJSPResult class (Listing 4) could be affected by the so-called *Middle Man* (Fowler and Beck 1999), i.e., a type of code smell that arises when a class delegates all its operations to other classes, hence uselessly increasing the complexity and computational costs of the system (Fowler and Beck 1999). In other terms, our analysis may suggest that the presence of code smells in production code may affect the test smell detection capabilities: the intrinsic relation between code and test smells is something we plan to explore as part of our future research agenda.

*Mystery Guest and Resource Optimism* When it turns to *Mystery Guest* and *Resource Optimism*, both are connected to the usage of external resources within a test method. By analyzing the reasons behind the detection failures, we could draw very similar conclusions:

*1. Inability to Handle Mocks* The use of mocks severely impacted the false positive rate of both test smells (respectively 893 and 1,202 cases) but for different reasons to those discussed for *Eager Test*. In particular, mocks create fake external dependencies that all the detectors mistakenly interpret as real. In the case of *Mystery Guest*, the detectors identified smelly instances because of those fake dependencies, which were not present. Instead, in the case of *Resource Optimism*, the detector could not detect any mechanism of verification of the existence/status of the resource, hence highlighting the presence of the smell: however, since mocks simulate the behavior of external resources, there is no need to verify their status, hence biasing the performance of all detectors.

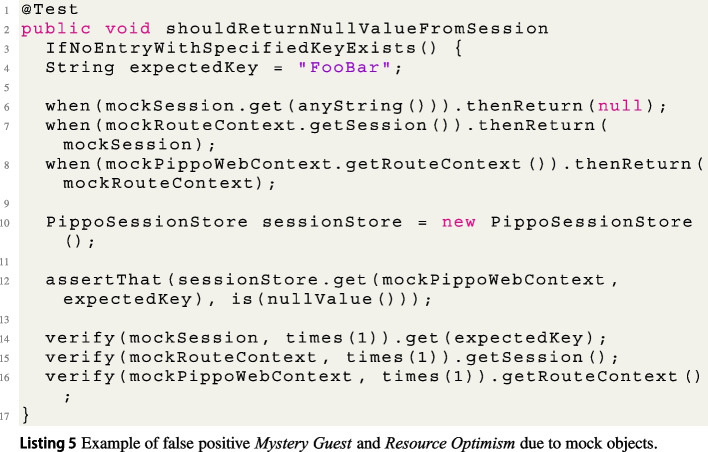



A representative example of false positive impacting the performance of both *Mystery Guest* and *Resource Optimism* detection is reported in Listing 5. As shown in the piece of code, the test shouldReturnNullValueFromSession of the project Pippo—a micro web framework for *Java*—makes significant use of mocking objects to simulate navigation session values. Such a dependency was therefore interpreted as a *Mystery Guest* instance. At the same time, the code does not check for the status of the mock; therefore, it was erroneously classified as a *Resource Optimism* instance. In conclusion, we could emphasize that mocking practices notably impact the performance of test smell detectors and that, therefore, novel mocking-aware detection strategies may provide significant contributions to the field.

*2. Incomplete operationalization of the definition* As for false negatives, we could identify a common reason for failure: the incomplete operationalization of the definitions of *Mystery Guest* (919 test cases) and *Resource Optimism* (453 test cases). Both smells arise when handling external resources (Van Deursen et al. 2001): yet, the definition does not provide a comprehensive list of what should be considered as an external resource—Van Deursen et al. (2001) just made the examples of files and databases. We suppose that the original definition was left open on purpose to include other types of external resources. Nevertheless, it seems that most detectors based their own detection rules solely on managing external files and databases without identifying issues when handling other types of resources. Therefore, this issue impacted the number of false negatives. An example is shown in Listing 6.

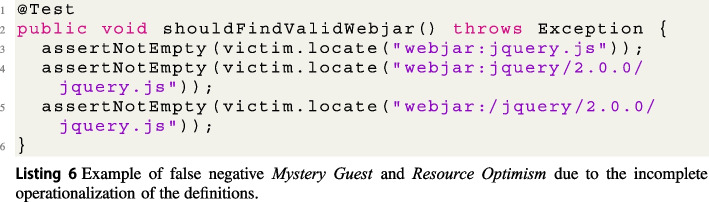



The example reports the case of the shouldFindValidWebjar test of the Wro4J project. The test checks if external JavaScript pages exist. All the detectors did not identify the external resource, overlooking this potential test smell. In conclusion, we argue that better detectors might be built by devising novel taxonomies to systematically collect comprehensive knowledge on how *Mystery Guest* and *Resource Optimism* instances may arise.

*Test Redundancy* The performance obtained by the experimented detectors on the *Test Redundancy* smell was close to 100% in terms of recall, meaning that they could detect all instances of the smell. However, the precision of the detectors was dramatically low, i.e., close to 0%. In this respect, there are two main points of discussion:

*1. Insufficient sample* As discussed in Section 4, our dataset contained very few instances of *Test Redundancy*. The low diffuseness of the smell was definitively one of the causes that let the machine learning-based approach fail: it was unable to learn the properties characterizing this test smell. In this sense, we may argue the need for alternative methods to feed machine learning-based approaches, e.g., defining synthetic training samples to complement the information provided by manually-validated instances.

*2. Lack of semantic redundancy analysis* The second critical threat to accurately detecting the smell was the inappropriate measurement performed by the current test redundancy metrics, which lack semantic analysis (identified in 43 test cases). Let us consider the example in Listing 7.

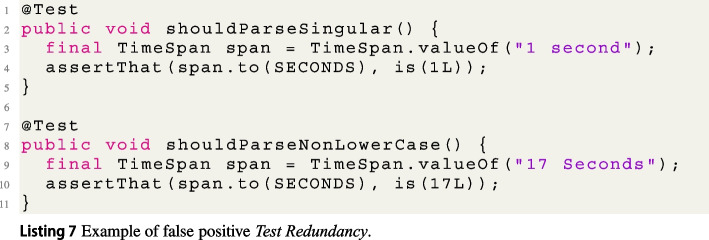



The example refers to the shoudParseSingular and shoudParseNonLower Case test cases of the Riptide project. These tests were identified as smelly by both TeReDetect and the machine learning-based approach. The test cases seem to exercise the same execution path, yet they do that in different manners. More specifically, the test cases aim at verifying the behavior of the valueOf method of the production class when this is supplied with timestamps expressed in seconds. While this case may look like an instance of *Test Redundancy*, it is worth considering that the values passed to the valueOf method have two very different meanings: shouldParseSingular exercises the production method with an extreme input (time cannot be negative; hence one second represents an extreme value of the input range of the production method), while shouldParseNonLowerCase with an in-range input (17 seconds). As such, the two methods cannot be considered redundant, as none of them can be removed without impacting the test suite - otherwise, developers would lose a relevant piece of information for the adequacy of the production code. Unfortunately, the pair redundancy metric exploited by the detectors only considers whether two test cases cover the same path without accounting for the rationale behind them. Therefore, we argue the need for more advanced metrics to combine dynamic and semantic analysis to discriminate redundancy cases correctly.

Concluding our argumentation on the root causes of test smell detection failures, we identified the current issues and challenges that researchers in the field are called to address in future research efforts. In addition, our analysis could shed lights on the limitations of currently available test smell detection tools: they indeed seem to rely on rather simple detection tactics that may fail in the wild because of the problems emerged from our analysis. Overall, we argue that the field of test smells would benefit from a systematic reinterpretation of its ground, which would more effectively inform the next generation of test smell detectors. This observation is especially true when considering contemporary testing practices, e.g., mocking, that naturally impact how test code quality is managed and assessed. It is our hope that the limitations of heuristic-based approaches highlighted by our work might stimulate researchers to devise novel, more robust and realistic detectors that might be resilient to the current issues.

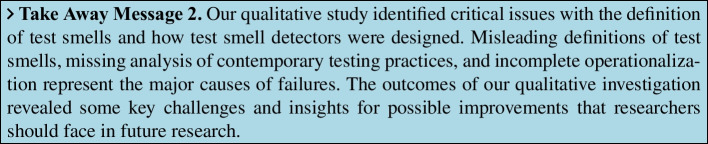



## Threats to Validity

Multiple factors might have biased the conclusions drawn in our empirical assessment. This section overviews the main limitations faced and how we mitigated them, discussing them based on their impact on our study.

*Construct Validity* When considering the relationship between theory and observation, the first potential limitation to discuss is concerned with the test smell dataset we relied on. In our research, we contributed a novel, manually-validated dataset composed of 9,633 test cases labeled according to their smelliness. We opted for constructing a novel dataset as, to the best of our knowledge, the current literature does not provide a sufficiently large dataset to experiment with machine learning algorithms. We approached the dataset construction through a formal validation procedure that involved multiple inspectors, who were called to label the smelliness of test methods available in the well-known IDoFT dataset. The inspectors interleaved manual validation sessions with open discussions of their actions to find a consistent procedure to assess the smelliness of the artifacts considered. In addition, the inspectors constantly monitored their agreement to tune the validation process. Since the process could still suffer from subjectivity, we performed a further step ahead by running a coherence check that involved real-world developers who were asked to validate—using a similar process as the inspectors—part of the test methods of the dataset. This additional step was performed to assess the potential subjectivity bias affecting the internal validation of test smells and measure how much our manual validation would align with the one performed by experienced developers. The external validation results were positive (Cohen’s $\kappa$=0.67) and indicated a good level of agreement (McHugh 2012). On the one hand, this allowed us to establish the overall soundness of the manual validation process. On the other hand, the lack of a full agreement was expected, as the validation of test smells has an intrinsically subjective nature. For this reason, it would have been nearly impossible to build a dataset that fully represents the perspective of a generic developer. In any case, we publicly released the dataset as part of our online appendix (Pontillo et al. 2023); further researchers may want to contribute to its understanding, improvement, and evolution.

A second limitation concerns how we computed the independent variables, i.e., the features considered by the machine learning solutions and heuristic approaches experimented with in the study. We specifically collected and relied on the metrics previously defined in test smell research. First, this choice allowed us to set a fair comparison between machine learning-based test smell detectors and heuristic approaches. Second, the definition of novel metrics was outside the scope of our study, as this would have required their preliminary theoretical and empirical evaluations (Fenton and Bieman 2014). Nonetheless, as part of our further analyses, we attempted to identify the limitations of current metrics to provide researchers with insights into the next steps that might be performed to improve test smell detection.

*Conclusion Validity* As for the limitations due to the relation between treatment and outcome, a key potential source of bias may have been related to the presence of independent variables providing a similar contribution to the performance of the experimented machine learning models: it has indeed been shown that this situation may increase noise when training a machine learning algorithm, finally biasing its performance (O’brien 2007). To account for this potential threat, we purposely defined **RQ**_1_ to probe each feature of the models, i.e., we computed the information gain provided by each feature used to feed the models (Quinlan 1986). Such a process allowed us to verify that the independent variables were orthogonal, contributing to the models built. Along the same line, another discussion point concerns the possible noise caused by specific pre-processing steps applied when building the machine learning pipeline. In this respect, we opted for an *ablation* study (Lipton and Steinhardt 2019) through which we could assess the contribution of each pre-processing step, hence identifying the best pipeline configuration to use in our study.

We did not have a baseline for machine learning algorithms experimented with, as our work represents the first attempt to study machine learning for test smell detection. As such, we experimented with multiple techniques to identify the best algorithm. For the sake of readability, we did not discuss all the results in Section 6; yet, our online appendix (Pontillo et al. 2023) includes all our findings, which researchers can use to understand further the impact of machine learning techniques on the performance of test smell detection.

In the context of **RQ**_2_, we assessed test smell detectors based on machine learning under two different use case scenarios, considering both within- and cross-project training. This analysis was done to increase the scope of our analysis and provide insights into the capabilities of machine learning in different contexts. We relied on well-established validation approaches such as cross-fold (Stone 1974) and leave-one-project-out validation (Refaeilzadeh et al. 2009). To further corroborate the conclusions drawn in the study, we finally applied the Nemenyi test (Nemenyi 1963), which allowed us to report our findings from a statistical perspective.

*External Validity* As for the generalizability of the conclusions, the dataset exploited was based on open-source projects written in *Java*. We cannot, therefore, ensure that our findings hold when considering different programming languages or types of software systems. In this regard, further replications would still be desirable: for instance, recent efforts have been made to devise test smell detectors working on Python code (Vavrová and Zaytsev 2017; Wang et al. 2021). To stimulate further research, we made all our scripts available in our appendix (Pontillo et al. 2023).

## Conclusion

The ultimate goal of our work was to experiment with machine learning algorithms for test smell detection, relying on the set of features previously defined to characterize the source of test smells. In the first place, we defined a novel, publicly-available dataset of test smells, which we later used to feed a machine-learning pipeline. We investigated the performance of the devised machine learning solution in the context of an empirical study, where we assessed (1) the features that most contribute to the prediction of test smells; (2) the performance of 28,248 and 14,256 different configurations of machine learning pipelines in within- and cross-project training scenarios, respectively; and (3) how machine learning approaches compare to standard, heuristic-based test smell detectors. Our findings reported a negative result: none of the experimented machine learning pipelines reached an F-Measure higher than 51%, even though a machine learning approach often outperforms the heuristic-based techniques.

We did not limit ourselves to reporting on the negative result but also performed additional qualitative investigations aimed at (1) assessing the performance of the machine learning-based test smell detector when compared to dummy classifiers to provide a more pragmatical view of the performance of the detector, and (2) classifying the root-causes of failures that prevent test smell detectors from identifying test smell instances correctly. The additional insights of our study let emerge several open issues and challenges that the research community should address through future research.

To sum up, our paper provided the following contributions:

1. A novel publicly-available dataset of manually-validated test smell instances, which researchers may use to analyze test smells further;
2. An empirical investigation into the capabilities of machine learning approaches for test smell detection, which researchers can use as a baseline to build additional research on the matter;
3. A catalog of root causes of failures for test smell detection, which provides qualitative insights and practical examples of how the field of test smell detection could be improved to better support practitioners;
4. An online appendix (Pontillo et al. 2023) that contains all data and scripts used in the empirical study, which can be employed to replicate and extend ours.

The main considerations and conclusions of the study represent the input of our future research agenda. We will work toward better understanding and conceptualizing the test smell definitions and designing novel features that may better capture the concept of test smells. Furthermore, we plan to investigate the extent to which different machine learning and natural language processing techniques might empower test smell detection. In addition, we plan to analyze which software project characteristics could help select the most suitable approach for future work, considering that they should characterize the test code, the application code, and the development process. Eventually, such an analysis could lead to a meta-classifier to predict the most suitable detector. We also plan to elaborate on how design issues in production code may affect the performance of test smell detectors. Finally, to corroborate our findings, we plan to replicate our work in different contexts, e.g., on *Python* code.

## Data Availability

The manuscript has data included as electronic supplementary material. In particular: datasets generated and analyzed during the current study, detailed results, as well as scripts and additional resources useful for reproducing the study, are available as part of our online appendix on GitHub: https://github.com/darioamorosodaragona-tuni/ML-Test-Smell-Detection-Online-Appendix.

## Footnotes

1 Available at https://github.com/acmsigsoft/EmpiricalStandards.

2 https://mir.cs.illinois.edu/flakytests/

3 Prolific website: https://www.prolific.co/.

4 https://www.r-project.org/

5 The *Mockito* framework: https://site.mockito.org.

## References

[CR1] Aljedaani W, Peruma A, Aljohani A, Alotaibi M, Mkaouer MW, Ouni A, Newman CD, Ghallab A, Ludi S (2021) Test smell detection tools: a systematic mapping study. Eval Assess Softw Eng 170–180

[CR2] Antoniol G, Canfora G, Casazza G, De Lucia A, Merlo E (2002). Recovering traceability links between code and documentation. IEEE Trans Softw Eng.

[CR3] Azeem MI, Palomba F, Shi L, Wang Q (2019) Machine learning techniques for code smell detection: a systematic literature review and meta-analysis. Inf Softw Technol

[CR4] Baeza-Yates R, Ribeiro BdAN et al (2011) Modern information retrieval. New York: ACM Press; Harlow, England: Addison-Wesley

[CR5] Baldi P, Brunak S, Chauvin Y, Andersen CA, Nielsen H (2000). Assessing the accuracy of prediction algorithms for classification: an overview. Bioinformatics.

[CR6] Bavota G, Qusef A, Oliveto R, De Lucia A, Binkley D (2012) An empirical analysis of the distribution of unit test smells and their impact on software maintenance. In: 2012 28th IEEE international conference on software maintenance. IEEE, pp 56–65

[CR7] Bavota G, Qusef A, Oliveto R, De Lucia A, Binkley D (2015). Are test smells really harmful? An empirical study. Empir Softw Eng.

[CR8] Beck K (2003) Test-driven development: by example. Addison-Wesley Professional

[CR9] Beller M, Gousios G, Zaidman A (2017) Oops, my tests broke the build: an explorative analysis of Travis ci with Github. In: International conference on mining software repositories (MSR). IEEE, pp 356–367

[CR10] Bergstra J, Bengio Y (2012). Random search for hyper-parameter optimization. J Mach Learn Res.

[CR11] Breiman L (2001). Random forests. Mach Learn.

[CR12] Catolino G, Di Nucci D, Ferrucci F (2019) Cross-project just-in-time bug prediction for mobile apps: an empirical assessment. In: International conference on mobile software engineering and systems. IEEE, pp 99–110

[CR13] Catolino G, Ferrucci F (2019) An extensive evaluation of ensemble techniques for software change prediction. J Softw Evol Process e2156

[CR14] Catolino G, Palomba F, De Lucia A, Ferrucci F, Zaidman A (2018). Enhancing change prediction models using developer-related factors. J Syst Softw.

[CR15] Chawla NV, Bowyer KW, Hall LO, Kegelmeyer WP (2002). Smote: synthetic minority over-sampling technique. J Artif Intell Res.

[CR16] Cohen J (1960). A coefficient of agreement for nominal scales. Educ Psychol Measur.

[CR17] De Bleser J, Di Nucci D, De Roover C (2019) Assessing diffusion and perception of test smells in scala projects. In: International conference on mining software repositories. IEEE Press, pp 457–467

[CR18] De Bleser J, Di Nucci D, De Roover C (2019) Socrates: Scala radar for test smells. In: ACM SIGPLAN symposium on Scala. ACM, pp 22–26

[CR19] Di Nucci D, Palomba F, De Rosa G, Bavota G, Oliveto R, De Lucia A (2017) A developer centered bug prediction model. IEEE Trans Softw Eng

[CR20] Duda RO, Hart PE (1973). Pattern classification and scene analysis.

[CR21] Fenton N, Bieman J (2014) Software metrics: a rigorous and practical approach. CRC Press

[CR22] Fernandes E, Oliveira J, Vale G, Paiva T, Figueiredo E (2016) A review-based comparative study of bad smell detection tools. In: International conference on evaluation and assessment in software engineering. ACM, p 18

[CR23] Fowler M, Beck K (1999) Refactoring: improving the design of existing code. Addison-Wesley Professional

[CR24] Freund Y, Mason L (1999) The alternating decision tree learning algorithm. In: icml, vol 99. Citeseer, pp 124–133

[CR25] Garousi V, Küçük B (2018). Smells in software test code: a survey of knowledge in industry and academia. J Syst Softw.

[CR26] Gousios G, Zaidman A, Storey M, Van Deursen A (2015) Work practices and challenges in pull-based development: the integrator’s perspective. In: International conference on software engineering, vol 1. IEEE Press, pp 358–368

[CR27] Grano G, Palomba F, Di Nucci D, De Lucia A, Gall HC (2019). Scented since the beginning: on the diffuseness of test smells in automatically generated test code. J Syst Softw.

[CR28] Grano G, Palomba F, Gall HC (2019) Lightweight assessment of test-case effectiveness using source-code-quality indicators. IEEE Trans Softw Eng

[CR29] Greiler M, Van Deursen A, Storey MA (2013) Automated detection of test fixture strategies and smells. In: Software testing, verification and validation (ICST), pp 322–331

[CR30] Haiduc S, Bavota G, Oliveto R, De Lucia A, Marcus A (2012) Automatic query performance assessment during the retrieval of software artifacts. In: Proceedings of the 27th IEEE/ACM international conference on automated software engineering, pp 90–99

[CR31] Han H, Wang W, Mao B (2005) Borderline-smote: a new over-sampling method in imbalanced data sets learning. In: International conference on intelligent computing. Springer, pp 878–887

[CR32] Harrold MJ, McGregor JD, Fitzpatrick KJ (1992) Incremental testing of object-oriented class structures. In: Proceedings of the 14th international conference on software engineering, pp 68–80

[CR33] He H, Bai Y, Garcia EA, Li S (2008) Adasyn: adaptive synthetic sampling approach for imbalanced learning. In: International joint conference on neural networks (IEEE world congress on computational intelligence). IEEE, pp 1322–1328

[CR34] Heckman JJ (1990) Selection bias and self-selection. In: Econometrics. Springer, pp 201–224

[CR35] Koochakzadeh N, Garousi V (2010) A tester-assisted methodology for test redundancy detection. Advan Softw Eng 2010

[CR36] Kramer O (2016) Scikit-learn. In: Machine learning for evolution strategies. Springer, pp 45–53

[CR37] Kruchten P, Nord RL, Ozkaya I (2012). Technical debt: from metaphor to theory and practice. IEEE Softw.

[CR38] Lambiase S, Cupito A, Pecorelli F, De Lucia A, Palomba F (2020) Just-in-time test smell detection and refactoring: the darts project. In: International conference on program comprehension, pp 441–445

[CR39] Lipton ZC, Steinhardt J (2019). Troubling trends in machine learning scholarship: some ml papers suffer from flaws that could mislead the public and stymie future research. Queue.

[CR40] Mackinnon T, Freeman S, Craig P (2000) Endo-testing: unit testing with mock objects. Extreme Program Examined 287–301

[CR41] Maier F, Felderer M (2023) Detection of test smells with basic language analysis methods and its evaluation. In: 2023 IEEE international conference on software analysis, evolution and reengineering (SANER). IEEE, pp 897–904

[CR42] Maldonado EdS, Shihab E (2015) Detecting and quantifying different types of self-admitted technical debt. In: International workshop on managing technical debt (MTD). IEEE, pp 9–15

[CR43] Marcus A, Poshyvanyk D (2005) The conceptual cohesion of classes. In: International conference on software maintenance. IEEE, pp 133–142

[CR44] Martins L, Costa H, Machado I (2023) On the diffusion of test smells and their relationship with test code quality of java projects. J Softw Evol Process e2532

[CR45] McHugh ML (2012). Interrater reliability: the kappa statistic. Biochemia medica.

[CR46] McMinn P (2004). Search-based software test data generation: a survey. Softw Test Verification Reliab.

[CR47] Meszaros G (2007) xUnit test patterns: refactoring test code. Pearson Educ

[CR48] Myers GJ, Sandler C, Badgett T (2011) The art of software testing. John Wiley & Sons

[CR49] Nemenyi PB (1963) Distribution-free multiple comparisons. Princeton University

[CR50] Noble WS (2006). What is a support vector machine?. Nat Biotechnol.

[CR51] O’brien RM (2007) A caution regarding rules of thumb for variance inflation factors. Quality & Quantity 41(5):673–690

[CR52] Orso A, Silva S (1998) Open issues and research directions in object-oriented testing. In: Proceedings of the 4th international conference on achieving quality in software: software quality in the communication society (AQUIS’98)

[CR53] Palomba F, Di Nucci D, Panichella A, Oliveto R, De Lucia A (2016) On the diffusion of test smells in automatically generated test code: an empirical study. In: International workshop on search-based software testing. ACM, pp 5–14

[CR54] Palomba F, Zaidman A, De Lucia A (2018) Automatic test smell detection using information retrieval techniques. In: International conference on software maintenance and evolution. IEEE, pp 311–322

[CR55] Parizi RM, Lee SP, Dabbagh M (2014). Achievements and challenges in state-of-the-art software traceability between test and code artifacts. IEEE Trans Reliab.

[CR56] Pecorelli F, Di Lillo G, Palomba F, De Lucia A (2020) Vitrum: a plug-in for the visualization of test-related metrics. In: AVI 2020, pp 1–3

[CR57] Pecorelli F, Di Nucci D, De Roover C, De Lucia A (2019) On the role of data balancing for machine learning-based code smell detection. In: ACM SIGSOFT International workshop on machine learning techniques for software quality evaluation, pp 19–24

[CR58] Pecorelli F, Palomba F, Di Nucci D, De Lucia A (2019) Comparing heuristic and machine learning approaches for metric-based code smell detection. In: International conference on program comprehension. IEEE Press, pp 93–104

[CR59] Pedregosa F, Varoquaux G, Gramfort A, Michel V, Thirion B, Grisel O, Blondel M, Prettenhofer P, Weiss R, Dubourg V, Vanderplas J, Passos A, Cournapeau D, Brucher M, Perrot M, Duchesnay E (2011). Scikit-learn: machine learning in Python. J Mach Learn Res.

[CR60] Perez A, Abreu R, van Deursen A (2017) A test-suite diagnosability metric for spectrum-based fault localization approaches. In: International conference on software engineering. IEEE Press, pp 654–664

[CR61] Peruma A, Almalki K, Newman CD, M, MW, Ouni A, Palomba F (2020) Tsdetect: an open source test smells detection tool. In: ACM joint meeting on European software engineering conference and symposium on the foundations of software engineering, pp 1650–1654

[CR62] Pezzè M, Young M (2008) Software testing and analysis: process, principles, and techniques. John Wiley & Sons

[CR63] Pontillo V, Amoroso D’Aragona D, Pecorelli F, Di Nucci D, Ferrucci F, Palomba F (2023) Machine learning-based test smell detection — online appendix. https://github.com/darioamorosodaragona-tuni/ML-Test-Smell-Detection-Online-Appendix 10.1007/s10664-023-10436-2 PMC10914901 38456065

[CR64] Pontillo V, Palomba F, Ferrucci F (2021) Toward static test flakiness prediction: a feasibility study. In: International workshop on machine learning techniques for software quality evolution, pp 19–24

[CR65] Pontillo V, Palomba F, Ferrucci F (2022). Static test flakiness prediction: how far can we go?. Empir Softw Eng.

[CR66] Quinlan JR (1986). Induction of decision trees. Mach Learn.

[CR67] Qusef A, Bavota G, Oliveto R, Lucia AD, Binkley DW (2014). Recovering test-to-code traceability using slicing and textual analysis. J Syst Softw.

[CR68] Refaeilzadeh P, Tang L, Liu H (2009) Cross-validation. In: Encyclopedia of database systems. Springer, pp 532–538

[CR69] Rwemalika R, Habchi S, Papadakis M, Le Traon Y, Brasseur MC (2023). Smells in system user interactive tests. Empir Softw Eng.

[CR70] Sakshaug JW, Schmucker A, Kreuter F, Couper MP, Singer E (2016). Evaluating active (opt-in) and passive (opt-out) consent bias in the transfer of federal contact data to a third-party survey agency. J Survey Stat Method.

[CR71] Samarthyam G, Muralidharan M, Anna, RK (2017) Understanding test debt. In: Trends in software testing. Springer, pp 1–17

[CR72] Schapire RE (2013) Explaining adaboost. In: Empirical inference. Springer, pp 37–52

[CR73] Sheldon MR, Fillyaw MJ, Thompson WD (1996). The use and interpretation of the friedman test in the analysis of ordinal-scale data in repeated measures designs. Physiother Res Int.

[CR74] Spadini D, Palomba F, Baum T, Hanenberg S, Bruntink M, Bacchelli A (2019) Test-driven code review: an empirical study. In: International conference on software engineering. IEEE Press, pp 1061–1072

[CR75] Spadini D, Palomba F, Zaidman A, Bruntink M, Bacchelli A (2018) On the relation of test smells to software code quality. In: 2018 IEEE international conference on software maintenance and evolution. IEEE, pp 1–12

[CR76] Stone M (1974). Cross-validatory choice and assessment of statistical predictions. J Roy Stat Soc Ser B (Methodol).

[CR77] Taud H, Mas J (2018) Multilayer perceptron (mlp). In: Geomatic approaches for modeling land change scenarios. Springer, pp 451–455

[CR78] Tufano M, Palomba F, Bavota G, Di Penta M, Oliveto R, De Lucia A, Poshyvanyk D (2016) An empirical investigation into the nature of test smells. In: International conference on automated software engineering, pp 4–15

[CR79] Van Deursen A, Moonen L, van den Bergh A, Kok G (2001) Refactoring test code. In: International conference on extreme programming and flexible processes in software engineering (XP2001), pp 92–95

[CR80] Van Rompaey B, Demeyer S (2009) Establishing traceability links between unit test cases and units under test. In: 2009 13th European conference on software maintenance and reengineering. IEEE, pp 209–218

[CR81] Van Rompaey B, Du Bois B, Demeyer S, Rieger M (2007). On the detection of test smells: a metrics-based approach for general fixture and eager test. IEEE Trans Softw Eng.

[CR82] Vavrová N, Zaytsev V (2017) Does python smell like java? tool support for design defect discovery in python. arXiv:1703.10882

[CR83] Wang T, Golubev Y, Smirnov O, Li J, Bryksin T, Ahmed I (2021) Pynose: a test smell detector for python. In: 2021 36th IEEE/ACM international conference on automated software engineering (ASE). IEEE, pp 593–605

[CR84] Wohlin C, Runeson P, Höst M, Ohlsson MC, Regnell B, Wesslén A (2012) Experimentation in software engineering. Springer Science & Business Media

[CR85] Yen S, Lee Y (2006) Under-sampling approaches for improving prediction of the minority class in an imbalanced dataset. In: Intelligent control and automation. Springer, pp 731–740

[CR86] Zhang Y, Mesbah A (2015) Assertions are strongly correlated with test suite effectiveness. In: Joint meeting on foundations of software engineering. ACM, pp 214–224

